# Targeting Nuclear Receptor Coactivator SRC‐1 Prevents Colorectal Cancer Immune Escape by Reducing Transcription and Protein Stability of PD‐L1

**DOI:** 10.1002/advs.202310037

**Published:** 2024-07-02

**Authors:** Yilin Hong, Qiang Chen, Zinan Wang, Yong Zhang, Bei Li, Hanshi Guo, Chuanzhong Huang, Xu Kong, Pingli Mo, Nengming Xiao, Jianming Xu, Yunbin Ye, Chundong Yu

**Affiliations:** ^1^ State Key Laboratory of Cellular Stress Biology Innovation Center for Cell Biology School of Life Sciences Xiamen University Xiamen 361102 P. R. China; ^2^ Zhejiang Key Laboratory of Pathophysiology Department of Biochemistry and Molecular Biology Health Science Center Ningbo University Ningbo Zhejiang 315211 P. R. China; ^3^ Key Laboratory of Precision Medicine for Atherosclerotic Diseases of Zhejiang Province Affiliated First Hospital of Ningbo University Ningbo Zhejiang 315010 P. R. China; ^4^ Laboratory of Immuno‐Oncology Clinical Oncology School of Fujian Medical University Fujian Cancer Hospital Fuzhou 350014 P. R. China; ^5^ Cancer Research Center School of Medicine Xiamen University Xiamen 361102 P. R. China; ^6^ Department of Molecular and Cellular Biology Baylor College of Medicine Houston Texas 77030 USA

**Keywords:** colorectal cancer, immune escape, immunotherapy, PD‐L1, SRC‐1

## Abstract

Programmed death‐ligand 1 (PD‐L1) is overexpressed in multiple cancers and critical for their immune escape. It has previously shown that the nuclear coactivator SRC‐1 promoted colorectal cancer (CRC) progression by enhancing CRC cell viability, yet its role in CRC immune escape is unclear. Here, we demonstrate that SRC‐1 is positively correlated with PD‐L1 in human CRC specimens. SRC‐1 deficiency significantly inhibits PD‐L1 expression in CRC cells and retards murine CRC growth in subcutaneous grafts by enhancing CRC immune escape via increasing tumor infiltration of CD8^+^ T cells. Genetic ablation of SRC‐1 in mice also decreases PD‐L1 expression in AOM/DSS‐induced murine CRC. These results suggest that tumor‐derived SRC‐1 promotes CRC immune escape by enhancing PD‐L1 expression. Mechanistically, SRC‐1 activated JAK‐STAT signaling by inhibiting SOCS1 expression and coactivated STAT3 and IRF1 to enhance PD‐L1 transcription as well as stabilized PD‐L1 protein by inhibiting proteasome‐dependent degradation mediated by speckle type POZ protein (SPOP). Pharmacological inhibition of SRC‐1 improved the antitumor effect of PD‐L1 antibody in both subcutaneous graft and AOM/DSS‐induced murine CRC models. Taken together, these findings highlight a crucial role of SRC‐1 in regulating PD‐L1 expression and targeting SRC‐1 in combination with PD‐L1 antibody immunotherapy may be an attractive strategy for CRC treatment.

## Graphical abstract

SRC‐1 promotes CRC immune escape by enhancing PD‐L1 expression via multiple mechanisms: SRC‐1 enhances HDAC8 expression to inhibit SOCS1 expression, leading to JAK1‐STAT3 signaling pathway activation and PD‐L1 up‐regulation; SRC‐1 coactivates STAT3 and IRF1 to enhance PD‐L1 transcription; SRC‐1 stabilizes PD‐L1 protein by preventing SPOP binding to PD‐L1 for ubiquitination and degradation.

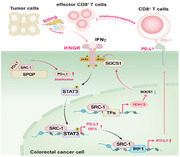

## 1 Introduction

Colorectal cancer (CRC), one of the common gastrointestinal tumors, is a leading cause of cancer mortality worldwide. Traditional cancer treatments have many side effects, high risk of metastasis and recurrence, and limited efficacy in patients with local or systemic metastasis.^[^ ^1^, ^2^ ^]^ In recent decades, the advent of tumor immunotherapy has revolutionized cancer treatment, which aims to eliminate malignant cells by stimulating or reconstructing the immune system;^[^ ^3^ ^]^ however, the immunosuppressive molecules expressed by cancer cells enable them to evade the surveillance of the immune system, which limits the efficacy of immunotherapy. Programmed death‐ligand 1 (PD‐L1) (also known as CD274) and programmed death‐1 (PD‐1)‐dependent pathways are essential immune checkpoints that have attracted extensive attentions. PD‐L1 expressed on solid tumor cells interacts with PD‐1 on tumor‐infiltrating lymphocytes (TILs) to impair the function and activation of immune cells and trigger tumor immune escape.^[^ ^4^, ^5^ ^]^ Overexpression of PD‐L1 is associated with immune escape and poor prognosis in tumor.^[^ ^6^ ^]^ Therefore, identification of distinct mechanisms in the modulation of PD‐L1 expression and function may provide a molecular basis to improve the clinical efficacy of tumor immunotherapy by targeting PD‐L1.

Genomic alterations, aberrant oncogenic signaling, exogenous factors and epigenetic mechanisms are closely related to the induction of tumor‐intrinsic expression of PD‐L1.^[^ ^5^, ^7^, ^8^ ^]^ Activation of Myc and Wnt/β‐catenin pathways up‐regulates PD‐L1 expression in multiple tumors.^[^ ^9^ ^]^ Genetic deletions of PTEN, a well‐known tumor suppressor gene, elevates the transcription of PD‐L1 via PI3K/AKT and β‐catenin/c‐Myc signaling pathway.^[^ ^10^, ^11^ ^]^ Interferon‐γ (IFNγ) is generally regarded as a dominant regulator of PD‐L1, and several tumor microenvironment (TME)‐resident cytokines such as IL‐1α and IL‐6 enhance PD‐L1 expression as well.^[^ ^12^ ^]^ Extensive research has shown that inflammatory cytokines‐related transcription factors including STAT1, STAT3, IRF1, and NF‐κB are involved in IFNγ‐induced PD‐L1 expression in different tumors.^[^ ^13^ ^]^ Consistently, our previous study showed that demethylase JMJD2D elevated PD‐L1 expression via activating IFNGR1‐STAT3‐IRF1 signaling in CRC.^[^ ^8^ ^]^

The transcription coactivator steroid receptor coactivator‐1 (SRC‐1) (also known as NCOA‐1) is a member in the SRC family that also contains SRC‐2 and SRC‐3. SRC‐1 has been strongly implicated in cancer progression and metastasis.^[^ ^14^ ^]^ Previous research has established that aberrantly elevated expression of SRC‐1 is observed in a variety of human cancer types, including CRC, prostate cancer, breast cancer, and thyroid cancer.^[^ ^15^, ^16^, ^17^ ^]^ As a coactivator, SRC‐1 not only coactivates with various nuclear receptors, such as estrogen receptor α (ERα), androgen receptor (AR), progesterone receptor (PR), glucocorticoid receptor (GR), and peroxisome proliferator‐activated receptor γ (PPARγ),^[^ ^18^ ^]^ but also interacts with numerous other transcription factors (TFs), such as AP‐1, STATs, NF‐κB, and p53.^[^ ^19^, ^20^ ^]^ SRC‐1 interacts with NRs or TFs to recruit other components to form a large coactivator complexe, leading to chromatin remodeling and transcriptional activation of specific target genes.^[^ ^17^ ^]^ However, no studies have investigated whether SRC‐1 interacts with specific TFs to regulate genes involved in tumor immune escape. Considering that coactivator TAZ, a component of the Hippo signaling pathway, is reported to transcriptionally activate PD‐L1 by interacting with the transcription factor TEAD to promote tumor immune escape in lung and breast cancer,^[^ ^21^ ^]^ and transcriptional coactivator MRTF‐A interacts with NF‐κB/p65 to activate the transcription of PD‐L1 in non‐small‐cell lung cancer (NSCLC) cells,^[^ ^22^ ^]^ it is interesting to know whether the transcription coactivator SRC‐1 is a key orchestrator that also transcriptionally activates PD‐L1.

Our previous studies have demonstrated that SRC‐1 acts as a coactivator to promote CRC progression through enhancing GLI2‐mediated Hedgehog signaling.^[^ ^16^ ^]^ Besides, SRC‐1 also promoted human hepatocellular carcinoma (HCC) progression by activating Wnt/β‐catenin signaling.^[^ ^23^ ^]^ However, the exact role of tumor cell‐expressed SRC‐1 in tumor immune escape has not been reported yet. In the current study, we found that SRC‐1 up‐regulated PD‐L1 expression to facilitate the immune escape of CRC cells in vitro and in vivo. SRC‐1 served as a coactivator for STAT3 and IRF1 to promote the transcription of PD‐L1. In addition, SRC‐1 inhibited the degradation of PD‐L1 by interfering with the binding between SPOP and PD‐L1. Furthermore, pharmacological inhibition of SRC‐1 enhanced the antitumor effect of PD‐L1 antibody in murine CRC models, indicating that SRC‐1 might be an attractive therapeutic target for CRC.

## 2 Results

### 2.1 Genetic Ablation of SRC‐1 Down‐Regulates PD‐L1 Expression and Attenuates Tumor Immune Escape

**Figure 1:**
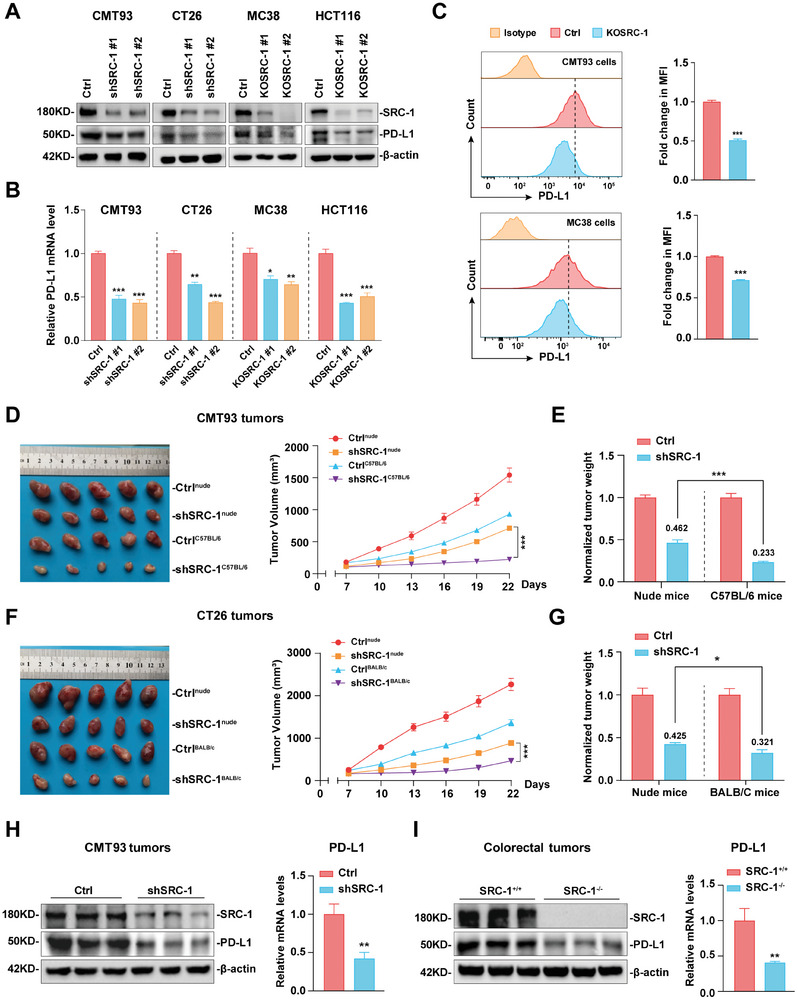
SRC‐1 up‐regulates PD‐L1 expression and promotes CRC immune escape. A, B) The protein (A) and mRNA (B, *n* = 3 per group) levels of PD‐L1 were attenuated in SRC‐1‐deficient CMT93, CT26, MC38, and HCT116 cells. C) FACS analysis showed that knockout of SRC‐1 reduced the membrane protein level of PD‐L1 in CMT93 and MC38 cells (*n* = 4 per group). D–G) The tumor burden of SRC‐1‐deficient CMT93 (D, E) and CT26 (F, G) cells in immunocompetent mice was lower than that in immunocompromised mice (*n* = 5 per group). Tumor weight was normalized with the mean value of the matched control group, and the percentage of tumor reduction in immunocompetent mice was higher than that in immunocompromised mice. H) The protein and mRNA (*n* = 4 per group) levels of PD‐L1 were attenuated in SRC‐1‐deficient CMT93 tumors. I) The protein and mRNA levels of PD‐L1 were reduced in AOM/DSS‐induced colorectal tumors of *SRC‐1* ^−/−^ mice (*n* = 5 per group). Xenograft tumor models are performed twice with similar results; other results are representative of one of three experiments. Data are shown as mean ± SEM. *p<0.05, **p<0.01, ***p<0.001, based on Student's t test, one‐way or two‐way ANOVA.

Several studies have demonstrated that SRC‐1 is highly expressed in multiple types of human solid cancers and plays a vital role in cancer cell initiation, progression, and metastasis by modulating different signaling pathways.^[^ ^24^, ^25^ ^]^ In addition, PD‐L1 is overexpressed in tumor cells, and binds to PD‐1 to inhibit the activation of immune cells, thereby mediating tumor immune escape. In view of this, we wonder whether SRC‐1 could regulate PD‐L1 expression in CRC. We knocked down or knocked out SRC‐1 in murine and human CRC cell lines, including CMT93, CT26, MC38, and HCT116. Western blot and RT‐qPCR analyses showed that the protein and mRNA levels of PD‐L1 were decreased in SRC‐1‐deficient CRC cells (**Figure** **1**A,B). Since mature PD‐L1 is anchored on the membrane after glycosylation modification to exert biological functions, the membrane level of PD‐L1 protein in CRC cells was detected using fluorescence activated cell sorting (FACS). The results showed that the membrane PD‐L1 protein levels were attenuated in SRC‐1‐deficient CMT93 and MC38 cells (Figure 1C). These results indicate that SRC‐1 is a positive regulator of PD‐L1 expression.

As SRC‐1 could positively regulate PD‐L1 expression, we speculated that SRC‐1 may promote CRC progression by up‐regulating PD‐L1 expression to enhance CRC immune escape. To test this possibility, the graft tumor models in immunocompromised nude mice and immunocompetent syngeneic mice were employed by inoculating the control (Ctrl) and SRC‐1‐deficient CMT93/CT26 cells, respectively. The growth and size of SRC‐1‐deficient tumors were significantly attenuated in both nude mice and immunocompetent mice compared to Ctrl tumors (Figure 1D,F), but the normalized weight of SRC‐1‐deficient tumors in immunocompetent mice was lower than that in nude mice (Figure 1E,G), suggesting that immune system in immunocompetent mice may contribute more to inhibit tumor growth after SRC‐1 down‐regulation in tumor. Consistent with in vitro results, the protein and mRNA levels of PD‐L1 were decreased in SRC‐1‐deficient CMT93 and CT26 tumors (Figure 1H; Figure S1A, Supporting Information). Similarly, the PD‐L1 level was also decreased in AOM/DSS‐induced primary colorectal tumors in *SRC‐1* ^−/−^ mice (Figure 1I), whose tumor burden was lower than that of *SRC‐1* ^+/+^ mice.^[^ ^16^ ^]^ Taken together, these results suggest that SRC‐1 may promote CRC immune escape by elevating PD‐L1 levels, thereby contributing to CRC progression.

### 2.2 SRC‐1 Deficiency in CRC Cells Promotes Tumor Infiltration and Antitumor Activity of CD8^+^ T Cells

**Figure 2:**
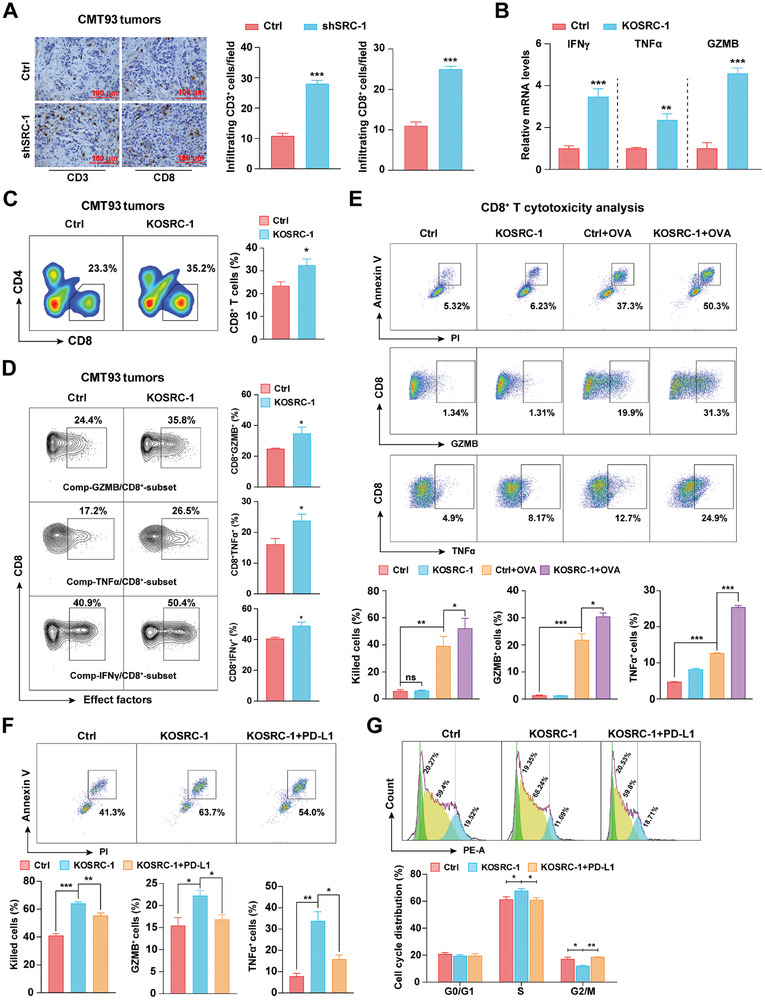
SRC‐1 promotes CRC cell immunosuppression by reducing tumor infiltration and antitumor activity of CD8^+^ T cells. A) Knockdown of SRC‐1 increased the number of infiltrated CD3^+^ and CD8^+^ T cells by immunohistochemical analysis in CMT93 tumors (*n* = 5 per group). B) The mRNA levels of IFNγ, GZMB, and TNFα were elevated in SRC‐1‐deficient CMT93 tumors (*n* = 4 per group). C) Representative FACS analysis plots and quantification of CD8^+^ T cells gated from CD45^+^ TILs in CMT93 tumors (*n* = 4 per group). D) Representative FACS analysis plots and quantification of the percentage of IFNγ^+^, GZMB^+^ and TNFα^+^ cells among CD8^+^ T cells in CMT93 tumors (*n* = 4 per group). E) The cytotoxicity and effector function of CD8^+^ T cells on SRC‐1‐deficient CMT93 cells were elevated (*n* = 3‐4 per group). The cell death of CMT93 and effector function of CD8^+^ T cells were evaluated by Annexin V/PI and GZMB/TNFα staining, respectively. F) The cytotoxicity and effector function of CD8^+^ T cells on exogenous PD‐L1 expression in SRC‐1‐deficient CMT93 cells were reduced compared with SRC‐1‐deficient group (*n* = 3 per group). The cell death of CMT93 and effector function of CD8^+^ T cells were evaluated by Annexin V/PI and GZMB/TNFα staining, respectively. G) Exogenous expression of PD‐L1 reversed the increased proportion of S‐phase CD8^+^ T cells in SRC‐1‐deficient CRC cells (*n* = 3 per group). Xenograft tumor models are performed twice with similar results; other results are representative of one of three experiments. Data are presented as means ± SEM. ^ns^ *p*>0.05, *p<0.05, **p<0.01, ***p<0.001, based on Student's t test or one‐way ANOVA.

Considering that the PD‐1 receptor of CD8^+^ T cells is a sensitive sensor of PD‐L1 expressed on the surface of tumor cells and it can trigger the self‐silencing of CD8^+^ T cells, we evaluated the infiltrating T cells in tumor tissues by employing the CD3/CD8 immunohistochemical staining. The results showed significantly increased infiltration of CD3^+^ and CD8^+^ T cells in SRC‐1‐deficient CMT93 and CT26 tumors (**Figure** **2**A; Figure S1B, Supporting Information), as well as increased mRNA levels of IFNγ, granzyme B (*GzmB*), and TNFα (Figure 2B), suggesting enhanced activity of infiltrating CD8^+^ T cells in SRC‐1‐deficient tumors. Furthermore, the effector T cell profiles of tumor infiltrating lymphocytes in CMT93 tumors were characterized using FACS. We found that the proportion of CD8^+^ T cells in SRC‐1‐deficient tumors was higher than that in Ctrl group (Figure 2C). What's more, the enrichment of effector T cells in CMT93 tumors was employed to further understand the role of tumor infiltrating cytotoxic T cells, and an increased percentage of IFNγ^+^CD8^+^, GZMB^+^CD8^+^ and TNFα^+^CD8^+^ T cells, which represent the activated and cytotoxicity of CD8^+^ T cells, were found in SRC‐1‐deficient tumors (Figure 2D). These results suggest that tumor cell‐expressed SRC‐1 promotes tumor immune escape by reducing tumor infiltration and vitality of CD8^+^ T cells.

To functionally assess whether tumor cell‐expressed SRC‐1 could inhibit the antitumor activity of CD8^+^ T cells, a co‐culture with effector CD8^+^ T cells and Ctrl or SRC‐1‐deficient CRC cells in vitro was performed, and then the tumor cell death and antitumor activity of CD8^+^ T cells were assessed by Annexin V/PI and GZMB/TNFα staining, respectively. The CD8^+^ T cells purified from OT‐I mice were activated by mIL‐2 and OVA^257‐264^ peptide,^[^ ^26^ ^]^ and then co‐cultured with OVA peptide‐labeled/unlabeled Ctrl and SRC‐1‐deficient CRC cells, respectively. The FACS analysis showed that the killing percentage of SRC‐1‐deficient tumor cells (KOSRC‐1+OVA) was higher than that of the corresponding Ctrl group (Figure 2E, upper), and the effector functions of CD8^+^ T cells in KOSRC‐1+OVA group were stronger as proved by the elevated levels of GZMB and TNFα in CD8^+^ T cells (Figure 2E, middle and lower). These results demonstrate that the immune resistance of SRC‐1‐deficient CRC cells to effector CD8^+^ T cells is attenuated.

To verify that reduced PD‐L1 is responsible for weakened immune resistance of SRC‐1‐deficient CRC cells to effector CD8^+^ T cells, we transfected PD‐L1 expression plasmid to rescue PD‐L1 expression in SRC‐1‐deficient CRC cells (Figure S1C, Supporting Information). Rescued PD‐L1 expression in SRC‐1‐deficient CRC cells partially recovered the immune resistance of SRC‐1‐deficient CRC cells to effector CD8^+^ T cells (Figure 2F; Figure S1D, Supporting Information). Additionally, cell cycle analysis showed an increase in S‐phase accumulation and a decrease in G2/M‐phase proportion of CD8^+^ T cells in SRC‐1‐deficient group was reversed by exogenous expression of PD‐L1 (Figure 2G). Taken together, these results suggest that tumor cell‐expressed SRC‐1 inhibits the antitumor activity of CD8^+^ T cells by up‐regulating PD‐L1 expression in tumor cells.

### 2.3 SRC‐1 Enhances PD‐L1 Expression by Promoting STAT3‐IRF1 Signaling via a Relief of JAK1 Inhibition

**Figure 3:**
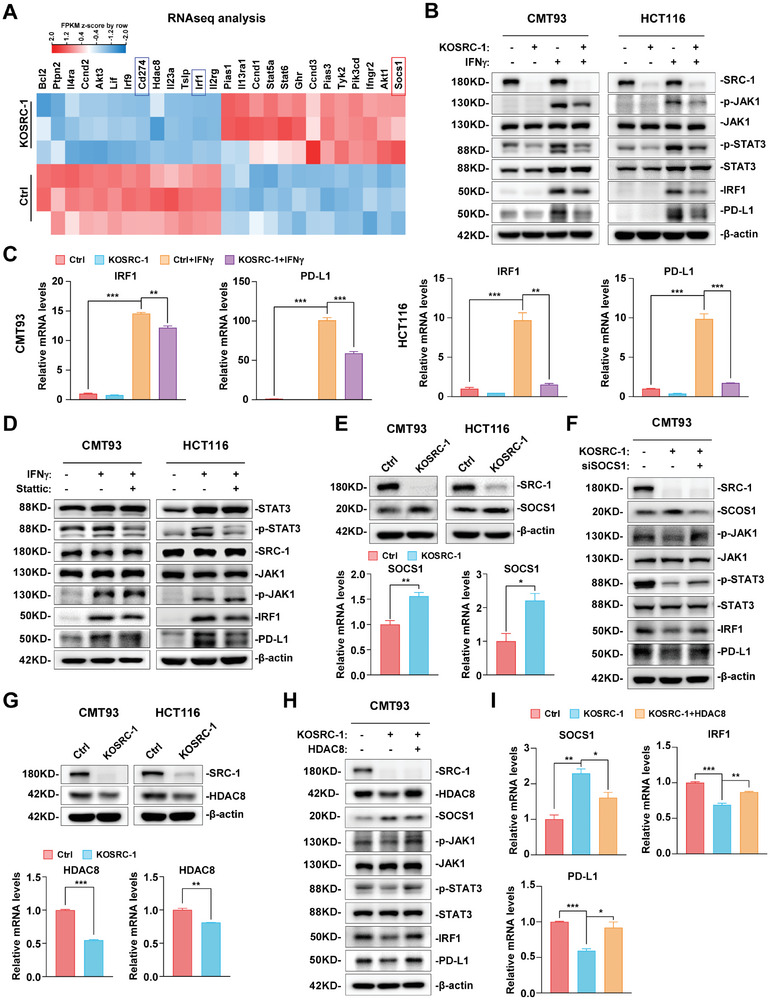
SRC‐1 enhances PD‐L1 expression by promoting STAT3‐IRF1 signaling via a relief of JAK1 inhibition. A) Heatmap of differentially expressed genes involved in JAK‐STAT pathway. The expression of CD274 (PD‐L1) and IRF1 were decreased in SRC‐1‐deficient MC38 cells. B) Knockout of SRC‐1 down‐regulated IFNγ‐induced phosphorylation levels of JAK1 and STAT3, as well as protein levels of IRF1 and PD‐L1 in CMT93 and HCT116 cells. C) The mRNA levels of IRF1 and PD‐L1 induced by IFNγ were decreased in SRC‐1‐deficient CMT93 and HCT116 cells (*n* = 3 per group). D) Stattic, a specific inhibitor of STAT3 phosphorylation, down‐regulated the protein levels of IRF1 and PD‐L1 in CMT93 and HCT116 cells. E) The protein and mRNA (*n* = 3 per group) levels of SOCS1 were up‐regulated in SRC‐1‐deficient CMT93 and HCT116 cells. F) Knockdown of SOCS1 in KOSRC‐1 CRC cells restored the phosphorylation of JAK1 and STAT3 as well as the expression of IRF1 and PD‐L1. G) The protein and mRNA (*n* = 3 per group) levels of HDAC8 were attenuated in SRC‐1‐deficient CMT93 and HCT116 cells. H, I) Exogenous expression of HDAC8 in KOSRC‐1 CRC cells down‐regulated SOCS1 expression and rescued JAK1‐STAT3 signaling and the protein and mRNA (*n* = 3 per group) levels of IRF1 and PD‐L1. Data are presented as means ± SEM. *p<0.05, **p<0.01, ***p<0.001, based on Student's t test or one‐way ANOVA.

To reveal the mechanism by which SRC‐1 regulates PD‐L1 expression, the COAD data in TCGA was employed to perform gene set enrichment analysis (GSEA). The JAK‐STAT signaling pathway, which is known to regulate PD‐L1 expression,^[^ ^12^ ^]^ was identified to be associated with SRC‐1 levels (Figure S2A, Supporting Information). Moreover, RNA‐seq analyses of Ctrl and SRC‐1‐knockout CRC cells showed that IRF1, a key transcription factor of PD‐L1, was significantly down‐regulated in KOSRC‐1 cells (**Figure** **3**A). Previous studies have shown that IRF1 expression is up‐regulated by the IFNγ‐activated JAK‐STAT signaling pathway.^[^ ^12^, ^27^ ^]^ Hence, we speculated that SRC‐1 may activate the JAK‐STAT signaling pathway to induce PD‐L1 expression. To test this, we treated CRC cells with IFNγ and investigated the relationship between SRC‐1 and JAK‐STAT signaling pathway. IFNγ treatment significantly elevated phosphorylated JAK1 (p‐JAK1) and phosphorylated STAT3 (p‐STAT3) levels (Figure 3B; Figure S2C, Supporting Information), leading to enhanced expression of IRF1 and PD‐L1 (Figure 3B,C; Figure S2B,C, Supporting Information); whereas genetic deletion of SRC‐1 significantly reduced IFNγ‐induced JAK1‐STAT3 signal activation and up‐regulation of IRF1 and PD‐L1 (Figure 3B,C; Figure S2B,C, Supporting Information). Furthermore, partial blockage of the JAK‐STAT signaling pathway by Stattic, a specific inhibitor of STAT3 phosphorylation, compromised IRF1 and PD‐L1 expression (Figure 3D; Figure S2D, Supporting Information), confirming that the expression of IRF1 and PD‐L1 is regulated by JAK1‐STAT3 signaling. These data suggest that genetic ablation of SRC‐1 attenuates IRF1 and PD‐L1 expression by weakening JAK1‐STAT3 signaling.

RNA‐seq analyses showed that the expression of suppressor of cytokine signaling 1 (SOCS1), which can deactivate the JAK‐STAT signaling pathway by inhibiting the kinase activity of JAK1,^[^ ^28^ ^]^ was up‐regulated in KOSRC‐1 CRC cells (Figure 3A). Furthermore, up‐regulation of SOCS1 in KOSRC‐1 CRC cells was confirmed by RT‐qPCR and Western blot analysis (Figure 3E). To verify the involvement of SOCS1 in suppressing JAK1‐STAT3 signaling in KOSRC‐1 CRC cells, we knocked down SOCS1 in wild‐type and KOSRC‐1 CMT93 cells and then analyzed the activity of JAK1‐STAT3 signaling and the expression of IRF1 and PD‐L1. Knockdown of SOCS1 in wild‐type CMT93 cells up‐regulated the phosphorylation of JAK1 and STAT3 as well as protein and mRNA levels of IRF1 and PD‐L1 (Figure S2E,F, Supporting Information), and knockdown of SOCS1 in KOSRC‐1 CMT93 cells partially restored p‐JAK1 and p‐STAT3 protein levels as well as IRF1 and PD‐L1 expression (Figure 3F), demonstrating that up‐regulation of SOCS1 in KOSRC‐1 CRC cells contributes to the suppression of JAK1‐STAT3 signaling.

It has been reported that histone deacetylase 8 (HDAC8) can inhibit SOCS1 expression,^[^ ^29^ ^]^ and our RNA‐seq data showed HDAC8 expression was down‐regulated in KOSRC‐1 CRC cells (Figure 3A), which was confirmed by RT‐qPCR and Western blot analysis (Figure 3G). Therefore, we speculated that SRC‐1 inhibited the expression of SOCS1 by modulating HDAC8 levels. Indeed, knockdown of HDAC8 in CMT93 cells could up‐regulate the protein and mRNA levels of SOCS1 (Figure S2G,H, Supporting Information). To further determine the effect of HDAC8 on SRC‐1‐mediated regulation of SOCS1 expression and JAK‐STAT signaling, we exogenously expressed HDAC8 in SRC‐1‐deficient CRC cells. We observed that restoring HDAC8 expression in KOSRC‐1 CRC cells down‐regulated SOCS1 expression and rescued JAK1‐STAT3 signaling and the protein and mRNA levels of IRF1 and PD‐L1 (Figure 3H,I). Collectively, these results suggest that SRC‐1 enhances PD‐L1 expression by promoting JAK1‐STAT3‐IRF1 signaling via HDAC8‐mediated down‐regulation of SOCS1.

### 2.4 SRC‐1 Cooperates with STAT3‐IRF1 Axis to Promote PD‐L1 Transcription

**Figure 4:**
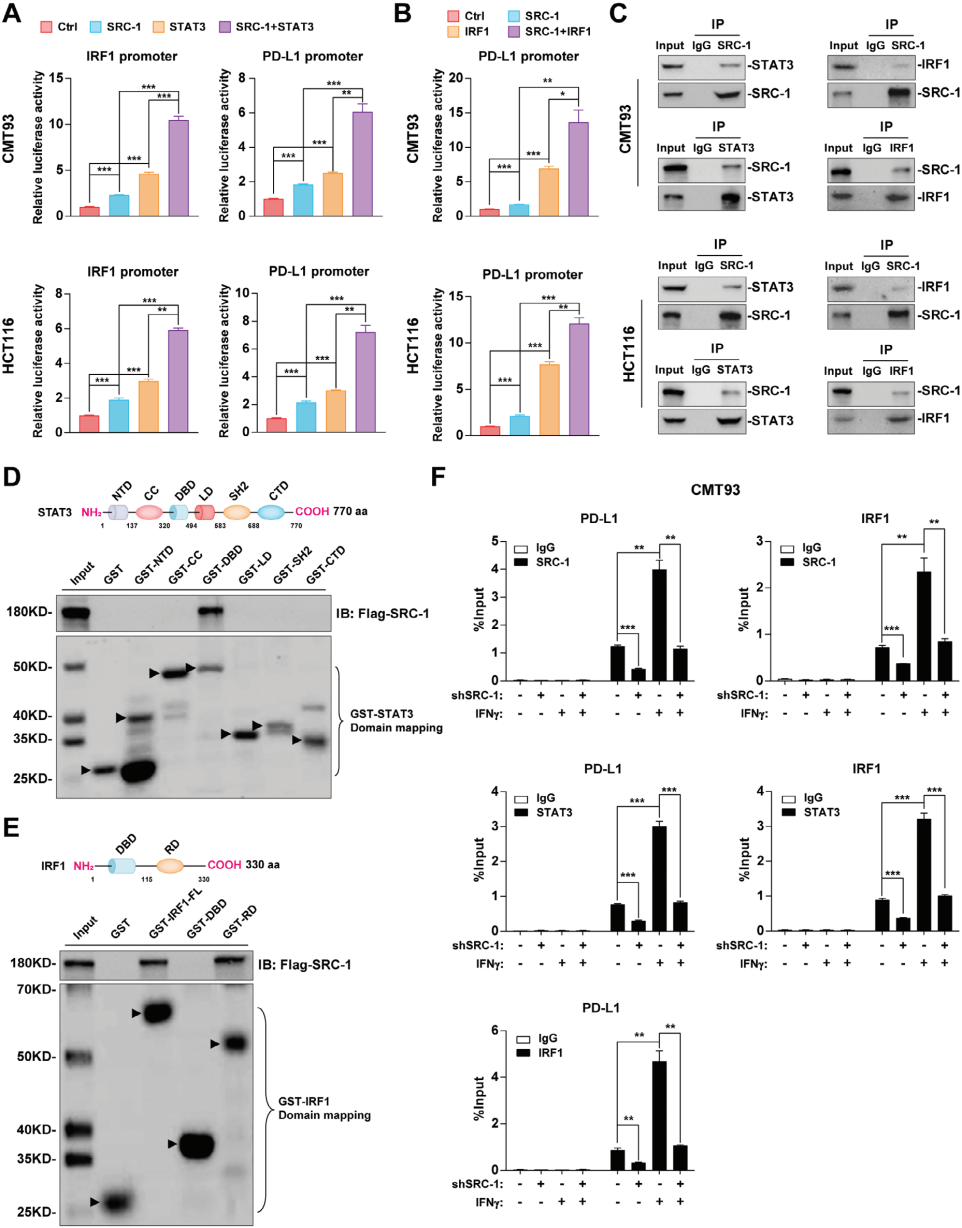
SRC‐1 cooperates with STAT3‐IRF1 axis to promote PD‐L1 transcription. A) SRC‐1 cooperated with STAT3 to enhance the transcriptional activity of IRF1 and PD‐L1 promoters in CMT93 and HCT116 cells (*n* = 3 per group). B) SRC‐1 cooperated with IRF1 to enhance the transcriptional activity of PD‐L1 promoter in CMT93 and HCT116 cells (*n* = 3 per group). C) The interaction between endogenous SRC‐1 and STAT3 or IRF1 was detected in CMT93 and HCT116 cells by Co‐IP assay. D, E) SRC‐1 could bind to the DBD domain of STAT3 (D) and RD domain of IRF1 (E), respectively. The arrow shows the representation strip. F) The recruitment of IFNγ‐driven STAT3 or IRF1 to their target gene promoters was prevented in CMT93 cells with SRC‐1 knockdown (*n* = 3 per group). Results are representative of one of three experiments. Data are presented as means ± SEM. *p<0.05, **p<0.01, ***p<0.001, based on one‐way ANOVA.

Our previous study has recognized the transcriptional coactivation role of SRC‐1 in CRC progression,^[^ ^16^ ^]^ and current study has indicated the modulation role of SRC‐1 in JAK1‐STAT3 signal transduction and IRF1 transcription (Figure 3). To determine whether SRC‐1 is a potential coactivator of the transcription factors STAT3 and IRF1 to drive the transcription of IRF1 and PD‐L1, the promoter reporters of IRF1 and PD‐L1 were constructed, and then their transcriptional activities were evaluated by dual luciferase reporter assay. The results showed that both SRC‐1 and STAT3 could independently promote the promoter activities of IRF1 and PD‐L1, whereas co‐transfection of SRC‐1 and STAT3 exhibited a synergistic effect (**Figure** **4**A; Figure S3A, Supporting Information), suggesting that SRC‐1 can cooperate with STAT3 to promote the transcription of IRF1 and PD‐L1. Similarly, co‐transfection of SRC‐1 and IRF1 exhibited a synergistic effect on the promotion of PD‐L1 transcription (Figure 4B; Figure S3B, Supporting Information), suggesting that SRC‐1 can cooperate with IRF1 to promote PD‐L1 transcription.

Furthermore, Co‐IP analysis demonstrated that SRC‐1 physically interacted with STAT3 and IRF1 in both CMT93 and HCT116 cells (Figure 4C). To investigate whether SRC‐1 can directly bind to STAT3 or IRF1, we conducted GST‐pull down assays. Domain mapping assays revealed that STAT3 interacted with the basic‐helix‐loop‐helix‐S/T (bHLH‐S/T) of SRC‐1 and SRC‐1 complexed with the DBD of STAT3 (Figure 4D; Figure S3C, Supporting Information). Similarly, the bHLH‐S/T of SRC‐1 and the regulation domain (RD) of IRF1 are responsible for their interaction (Figure 4E; Figure S3C, Supporting Information). Afterwards, ChIP assays were performed to determine whether SRC‐1 affects the recruitment of STAT3 or IRF1 to the promoters of their target genes. The enrichment of STAT3 at IRF1 and PD‐L1 promoters was evidently decreased in CMT93 (Figure 4F) and HCT116 (Figure S3D, Supporting Information) cells with SRC‐1 knockdown or knockout compared to the corresponding Ctrl cells; and similar results were obtained in experiments of IRF1 binding to the PD‐L1 promoter (Figure 4F; Figure S3D, Supporting Information), suggesting that SRC‐1 is essential for STAT3 and IRF1 recruitment. Collectively, these results indicate that SRC‐1 directly interacts with STAT3 or IRF1 to serve as a coactivator to promote their target gene transcription.

### 2.5 SRC‐1 Blocks E3 Ubiquitin Ligase SPOP‐Mediated Degradation of PD‐L1

**Figure 5:**
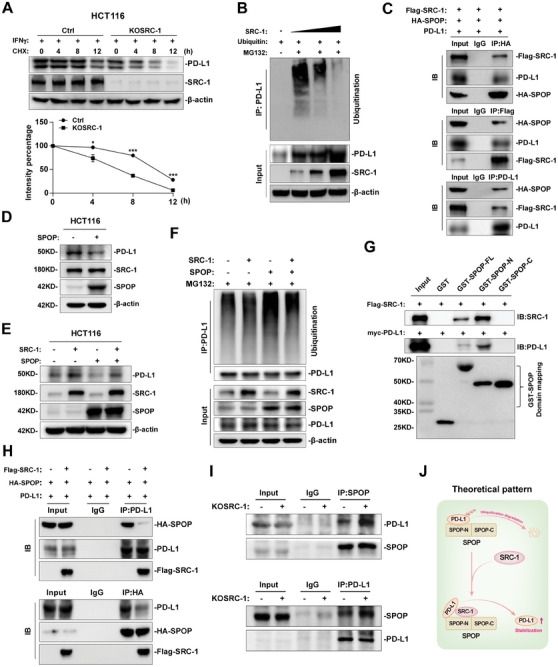
SRC‐1 blocks E3 ubiquitin ligase SPOP‐mediated degradation of PD‐L1. A) Knockout of SRC‐1 accelerated IFNγ‐induced PD‐L1 protein degradation in HCT116 cells. B) SRC‐1 inhibited the ubiquitination of PD‐L1 in a dose‐dependent manner in vitro. C) The interaction between SRC‐1, SPOP, and PD‐L1 was detected by Co‐IP assay. D) Overexpression of SPOP inhibited PD‐L1 expression, but did not affect SRC‐1 expression in HCT116 cells. E) SPOP‐mediated PD‐L1 degradation could be prevented by exogenous SRC‐1 in HCT116 cells. F) SRC‐1 prevented the poly‐ubiquitination modification of PD‐L1 by SPOP. G) SRC‐1 and PD‐L1 could interact with the N‐terminal domain of SPOP. H) The combination of SPOP and PD‐L1 could be blocked by exogenous SRC‐1. I) Knocking out SRC‐1 promotedc the interaction between SPOP and PD‐L1 in CRC cells. J) The theoretical pattern of SRC‐1 blocking SPOP‐mediated degradation of PD‐L1. Results are representative of one of three experiments. Data are presented as means ± SEM. *p<0.05, ***p<0.001, based on Student's t test.

In addition to the transcriptional regulation, protein stability regulation is also an important way for tumor cells to up‐regulate PD‐L1 level, in which the ubiquitin degradation mediated by ubiquitin proteasome system is critical.^[^ ^30^, ^31^ ^]^ Although our study has demonstrated that SRC‐1 transcriptionally regulates PD‐L1 expression through STAT3‐IRF1 axis, whether PD‐L1 is post‐transcriptionally regulated by SRC‐1 is unclear. The protein synthesis inhibitor cycloheximide (CHX) and the transcription inhibitor actinomycin D (Act D) were employed to investigate the post‐transcriptional regulation of PD‐L1, and reduced PD‐L1 protein levels were found in CMT93 and HCT116 cells treated with CHX or Act D (Figure S4A, Supporting Information), suggested that post‐translational modification is required for PD‐L1 stabilization. Protein stability analysis using CHX treatment revealed that knockdown or knockout of SRC‐1 could accelerate PD‐L1 protein degradation in CMT93 and HCT116 cells (**Figure** **5**A; Figure S4B–D, Supporting Information), whereas overexpression of SRC‐1 slowed the degradation of PD‐L1 protein in 293T cells (Figure S4E, Supporting Information). These data suggest that SRC‐1 can stabilize PD‐L1 protein. Subsequently, we investigated whether SRC‐1 stabilizes PD‐L1 protein by preventing PD‐L1 protein ubiquitination. We found that the accumulation of ubiquitinated‐PD‐L1 protein was reduced by exogenous SRC‐1 expression (Figure 5B; Figure S4F, Supporting Information), suggesting that SRC‐1 stabilizes PD‐L1 protein by preventing ubiquitin‐dependent degradation of PD‐L1 protein.

It has been reported that speckle‐type POZ protein (SPOP), an E3 ubiquitin ligase, mediates the ubiquitin‐dependent degradation of PD‐L1 protein.^[^ ^32^, ^33^ ^]^ The mRNA levels of SPOP in SRC‐1‐deficient HCT116 or CMT93 cells were invariable compared with the Ctrl cells (Figure S4G, Supporting Information), excluding the possibility that SRC‐1 inhibits SPOP expression to stabilize PD‐L1 protein. Since SPOP also interacts with SRC‐3 protein to mediate the ubiquitin‐dependent degradation of SRC‐3 protein,^[^ ^34^ ^]^ we speculated that SPOP may interact with SRC‐1 protein, considering high homolog between SRC‐1 and SRC‐3 proteins. As expected, Co‐IP analysis demonstrated that SPOP interacted with SRC‐1 and PD‐L1 proteins (Figure 5C). Intriguingly, overexpression of SPOP reduced PD‐L1 expression, but did not affect the expression of SRC‐1 in HCT116 cells (Figure 5D). Therefore, we speculated that SRC‐1 stabilizes PD‐L1 protein by binding to SPOP to prevent SPOP‐mediated ubiquitin‐dependent degradation of PD‐L1 protein. Indeed, when exogenous SRC‐1 and SPOP were expressed individually/jointly in HCT116 and 293T cells, exogenous SRC‐1 prevented SPOP‐mediated down‐regulation of PD‐L1 protein expression (Figure 5E; Figure S4H, Supporting Information). Furthermore, SRC‐1 was confirmed to prevent the poly‐ubiquitination modification of PD‐L1 by SPOP (Figure 5F). Notably, GST‐pull down assays indicated that SRC‐1, SPOP, and PD‐L1 can form SRC‐1/SPOP/PD‐L1 complexe in the presence of SPOP, further confirming that SPOP can interact with SRC‐1 and PD‐L1 (Figure S4I, Supporting Information). Besides, we found that both SRC‐1 and PD‐L1 interacted with the N‐terminal domain of SPOP (Figure 5G), which is responsible for binding to the substrate, indicating that SRC‐1 may stabilize PD‐L1 protein by interfering PD‐L1 binding to the N‐terminal domain of SPOP. Further studies verified that SRC‐1 overexpression reduced PD‐L1 binding to SPOP in 293T cells, while SRC‐1 knockout increased PD‐L1 interaction with SPOP in CRC cells (Figure 5H,I). Based on these results, we proposed a model in which SRC‐1 prevents SPOP‐mediated degradation of PD‐L1 protein by binding to SPOP to interfere with the interaction between SPOP and PD‐L1 (Figure 5J).

### 2.6 Genetic Depletion or Pharmacological Inhibition of SRC‐1 Enhances the Antitumor Effect of PD‐L1 Antibody

**Figure 6:**
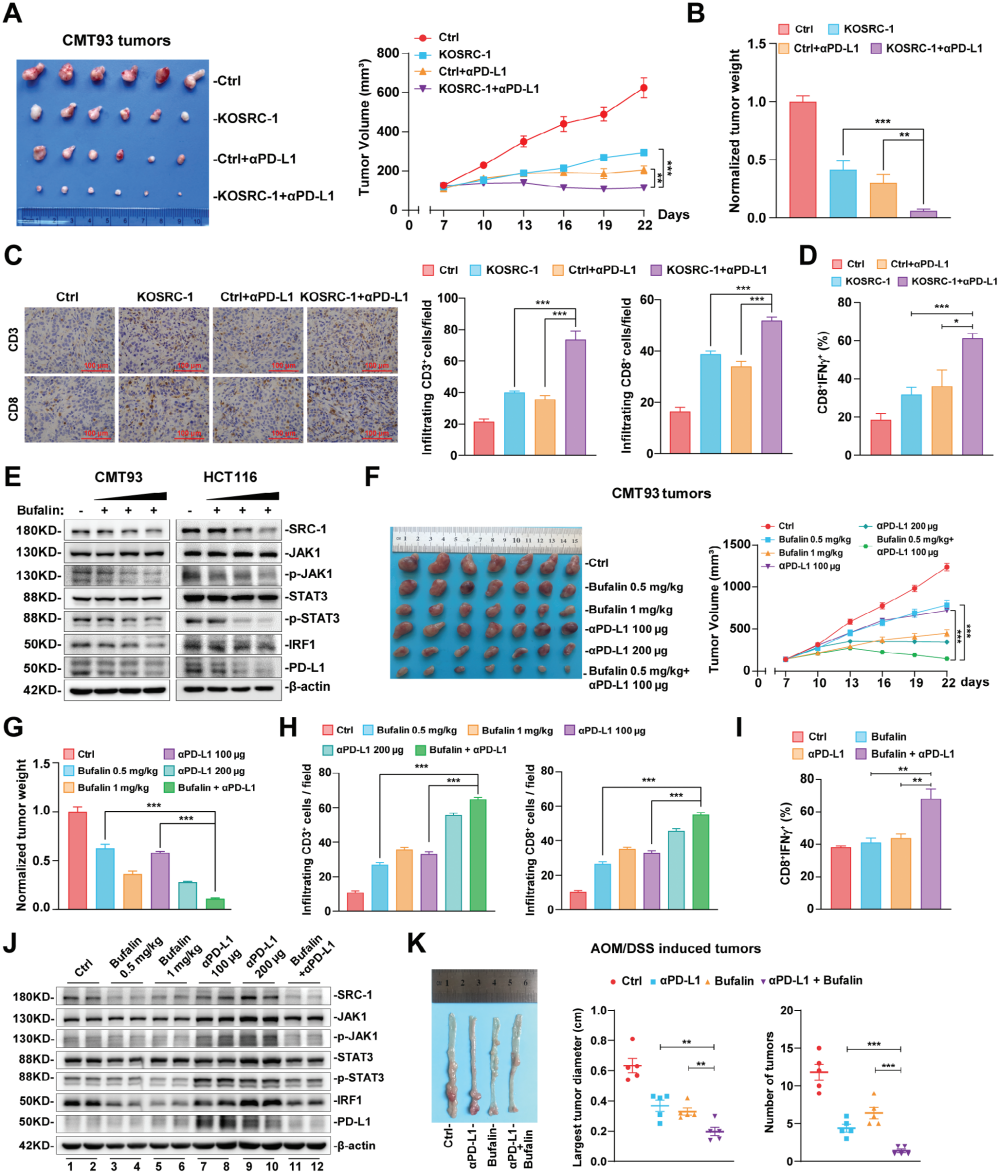
Genetic depletion or pharmacological inhibition of SRC‐1 enhances the antitumor effect of PD‐L1 antibody. A, B) The tumor growth (A) and weight (B) of CMT93 bearing mice were attenuated in the combination group compared with SRC‐1‐deficient or PD‐L1 antibody monotherapy at day 22 of treatment (*n* = 6 per group). Male C57BL/6 mice were subcutaneously inoculated with Ctrl or KOSRC‐1 CMT93 cells and treated with IgG control, PD‐L1 antibody (100 µg per mouse) and combination group. C) The number of tumor‐infiltrating CD3^+^ and CD8^+^ T cells in CMT93 tumors with indicated treatments (*n* = 6 per group) as described in (A, B). D) Quantification of IFNγ^+^ cells among CD8^+^ T cells in CMT93 tumors treated with SRC‐1 knockout and PD‐L1 antibody alone or in combination (*n* = 5 per group). E) SRC‐1 inhibitor bufalin suppressed JAK1‐STAT3‐IRF1 signaling and PD‐L1 expression in CMT93 and HCT116 cells in a dose‐dependent manner. F, G) The tumor growth (F) and weight (G) of CMT93 bearing mice were attenuated in the combination group compared to that of monotherapy with either low dose agent at day 22 of treatment (*n* = 7 per group). Male C57BL/6 mice were subcutaneously inoculated with CMT93 cells and treated with IgG control, bufalin (0.5 or 1 mg kg^−1^ per mouse), PD‐L1 antibody (100 or 200 µg per mouse) and combination group (bufalin 0.5 mg kg^−1^ and PD‐L1 antibody 100 µg per mouse). H) The number of tumor‐infiltrating CD3^+^ and CD8^+^ T cells in CMT93 tumors with indicated treatments (*n* = 5 per group) as described in (F, G). I) Quantification of IFNγ^+^ cells among CD8^+^ T cells in CMT93 tumors treated with bufalin and PD‐L1 antibody alone or in combination (*n* = 3 per group). J) Low dose combination treatment of bufalin and PD‐L1 antibody significantly inhibited JAK1‐STAT3 signaling and the expression of IRF1 and PD‐L1 in tumor tissues as described in (F, G). K) Low dose combination treatment of bufalin and PD‐L1 antibody decreased the size and number of AOM/DSS‐induced colorectal tumors (*n* = 5 per group). Tumor models are performed twice with similar results; other results are representative of one of three experiments. Data are presented as means ± SEM. *p<0.05, **p<0.01, ***p<0.001, based on one‐way or two‐way ANOVA.

Since SRC‐1 deficiency could reduce the expression of PD‐L1 in tumor cells, we speculated that SRC‐1 deficiency may enhance the antitumor activity of PD‐L1 antibody. We therefore compared the antitumor activity of PD‐L1 antibody on Ctrl and SRC‐1‐knockout CMT93 tumor‐bearing mice. The combination of SRC‐1 deficiency and PD‐L1 blockade elicited synergistic antitumor effects, manifested in a slower tumor growth, reduced tumor volume and weight compared with SRC‐1 deficiency alone or PD‐L1 antibody monotherapy group (**Figure** **6**A,B). In addition, SRC‐1 deficiency in CMT93 cells collaborates with PD‐L1 antibody to promote the number of tumor‐infiltrating CD3^+^ and CD8^+^ T cells (Figure 6C). Besides, FACS analysis of subcutaneous tumors revealed that SRC‐1 knockout combined with PD‐L1 antibody therapy also increased the effector function of tumor‐infiltrating CD8^+^ T cells, as reflected in the up‐regulation of IFNγ^+^CD8^+^ T cells proportion (Figure 6D; Figure S5A, Supporting Information).

Our previous study has identified the role of small molecule inhibitor bufalin in inhibiting the progression of CRC by suppressing SRC‐1 protein, while its role in inhibiting CRC immune escape is unclear. To this end, we treated CMT93 and HCT116 cells with a gradient concentration of bufalin, and found that pharmacological inhibition of SRC‐1 could significantly down‐regulate JAK1‐STAT3 signal and PD‐L1 level in a dose‐dependent manner (Figure 6E), indicating that bufalin has the potential to improve the efficacy of immunotherapy.

Immunotherapy with PD‐1/PD‐L1 blockade has shown low response rates in CRC patients due to insensitivity or resistance, and the combination of immunotherapy with targeting therapy may yield unexpected outcomes.^[^ ^35^, ^36^ ^]^ Thus, the graft tumor model of conditional processing was employed. CMT93 tumor‐bearing mice were treated according to the presupposition, including high and low doses of bufalin and PD‐L1 antibody, and a mixed strategy with low concentration. The results showed that the monotherapy of bufalin or PD‐L1 antibody stalled tumor growth, volume, and weight in a dose‐dependent manner, whereas the mixed strategy exhibited more powerful tumor suppression compared to monotherapy with either agent (Figure 6F,G). In addition, bufalin and PD‐L1 antibody combination treatment activated a potent antitumor immune response with the increase in the number (Figure 6H; Figure S5B, Supporting Information) and activity (Figure 6I; Figure S5C, Supporting Information) of tumor‐infiltrating CD8^+^ T cells, and eventually elicits prominent tumor growth inhibition. Intriguingly, we found that PD‐L1 antibody monotherapy could induce phosphorylation of JAK1 and STAT3, thereby up‐regulating the expression of IRF1 and PD‐L1 in tumor tissues (Figure 6J), which may also be one of the reasons why many patients are insensitive or resistant to PD‐L1 antibody monotherapy. Nevertheless, low‐dose combination of bufalin with PD‐L1 antibody exhibited a synergistic effect on the inhibition of JAK1‐STAT3 signal and the expression of IRF1 and PD‐L1 (Figure 6J).

The higher efficacy of the combined treatment was further confirmed in AOM/DSS‐induced CRC model, as indicated by the decrease of largest tumor diameter and tumor numbers (Figure 6K). Taken together, these data illustrate that SRC‐1 inhibitor bufalin promotes the antitumor efficacy of PD‐L1 antibody.

### 2.7 The Expression of SRC‐1 is Positively Correlated with PD‐L1 in Tumors of CRC Patients

**Figure 7:**
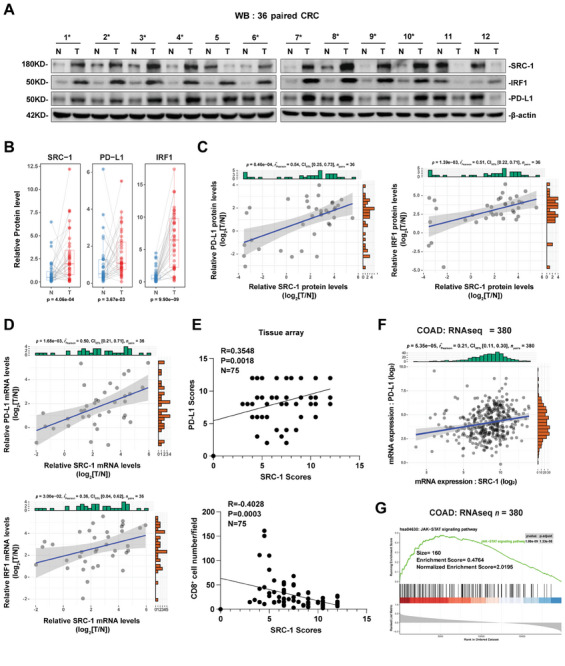
SRC‐1 is positively correlated with PD‐L1 in human CRC specimens. A) Protein levels of SRC‐1, IRF1, and PD‐L1 were measured in 12 out of 36 pairs of human CRC tumor tissues and their surrounding non‐tumorous tissues. The asterisk indicates SRC‐1‐positive CRC specimens. B) Relative protein levels of SRC‐1, IRF1, and PD‐L1 in human CRC samples (*n* = 36). Band density was quantified using ImageJ software and normalized to β‐actin levels. C) The protein level of SRC‐1 was positively correlated with IRF1 and PD‐L1 in human CRC specimens (*n* = 36). D) The mRNA level of SRC‐1 was positively correlated with IRF1 and PD‐L1 in human CRC specimens (*n* = 36). E) SRC‐1 was positively correlated with PD‐L1, but negatively correlated with the infiltration of CD8^+^ T cells in CRC tissue array specimens (*n* = 75). F) The positive correlation was found between SRC‐1 and PD‐L1 expression in COAD patients. G) The JAK‐STAT pathway was found to be associated with SRC‐1. The GSEA plot shows significant enrichment for the JAK‐STAT signaling pathway (normalized enrichment score (NES) = 2.02, and nominal *p* value = 1.88e^‐09^).

To reveal the clinical relevance of current study, we performed several feasible analyses of the expression profiles of SRC‐1, IRF1, and PD‐L1 in tumors or paracancerous tissues of 36 CRC patients. Western blot results showed that the protein levels of SRC‐1, IRF1, and PD‐L1 in tumor tissues of most patients were higher than those of paracancerous tissues (**Figure** **7**A,B; Figure S6, Supporting Information). Correlation analysis displayed that the protein and mRNA levels of SRC‐1 were positively correlated with IRF1 and PD‐L1 (Figure 7C,D). Our previous studies have characterized the profiles of CD8^+^ T cells infiltration and the expression of SRC‐1 and PD‐L1 in tissue arrays of 75 human CRC specimens.^[^ ^8^, ^16^ ^]^ After re‐integration and re‐analysis of these data, we found that SRC‐1 was positively correlated with PD‐L1, but negatively correlated with the infiltration of CD8^+^ T cells (Figure 7E), suggesting that SRC‐1 prevents CD8^+^ T cells infiltration in CRC by up‐regulating PD‐L1 expression.

Similarly, a positive correlation between SRC‐1 and PD‐L1 was found by analyzing RNA‐seq data of COAD in TCGA (Figure 7F); the dataset subsequently underwent GSEA analysis, and the JAK‐STAT pathway was found to be associated with SRC‐1 (Figure 7G). Intriguingly, the positive correlation between SRC‐1 and PD‐L1 and notable enrichment of JAK‐STAT pathway were also found in numerous non‐CRC cancer cell panels, including LIHC, PAAD, PRAD, CHOL, KIRC, THYM, etc. (Figure S7A–C, Supporting Information), suggesting that regulation of PD‐L1 by SRC‐1 may be applicable to various cancers.

## 3 Discussion

**Figure 8:**
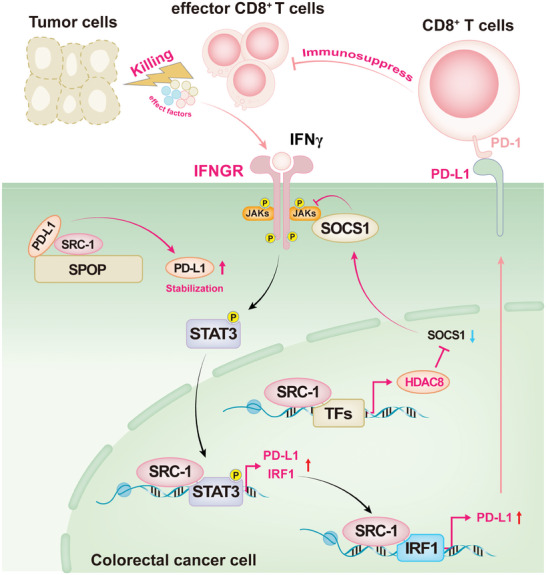
Schematic working model for the mechanisms by which SRC‐1 promotes CRC immune escape by elevating PD‐L1 transcription and protein stabilization. SRC‐1 enhances PD‐L1 expression via multiple mechanisms: 1) SRC‐1 enhances HDAC8 expression to inhibit SOCS1 expression, leading to JAK1‐STAT3 signaling pathway activation and PD‐L1 up‐regulation; 2) SRC‐1 coactivates STAT3 and IRF1 to enhance PD‐L1 transcription; 3) SRC‐1 stabilizes PD‐L1 protein by preventing SPOP binding to PD‐L1 for ubiquitination and degradation.

In the current study, we demonstrate that SRC‐1 promotes CRC immune escape by up‐regulating PD‐L1 expression in CRC cells. Multiple mechanisms by which SRC‐1 enhances PD‐L1 expression are presented in **Figure** **8**: 1) SRC‐1 inhibits SOCS1 expression via HDAC8 induction to allow relief of direct repression of JAK1‐STAT3 signaling pathway by SOCS1, leading to JAK1‐STAT3 signaling pathway activation and PD‐L1 up‐regulation; 2) SRC‐1 coactivates STAT3 and IRF1 to enhance PD‐L1 expression; 3) SRC‐1 stabilizes PD‐L1 protein by preventing SPOP binding to PD‐L1 for ubiquitination and degradation.

In this study, we showed that SRC‐1 activated JAK‐STAT signaling pathway to induce PD‐L1 expression. In addition to JAK‐STAT, several signaling pathways such as Mitogen‐activated protein kinase (MAPK), Wnt/β‐catenin, Hedgehog, and HIF1 can enhance PD‐L1 expression.^[^ ^8^, ^37^, ^38^ ^]^ Considering that SRC‐1 could also activate Wnt/β‐catenin, Hedgehog, and HIF1 signaling pathways,^[^ ^16^, ^23^, ^39^ ^]^ SRC‐1 may enhance the expression of PD‐L1 through these signaling pathways.

In addition to transcriptional regulation, the PD‐L1 level in tumor cells is also regulated by PTMs, including ubiquitination, glycosylation, phosphorylation, palmitoylation, SUMOylation, and acetylation.^[^ ^40^ ^]^ It has been reported that glycogen synthase kinase 3β (GSK3β) could interact with PD‐L1 and induce phosphorylation‐dependent proteasome degradation of PD‐L1 by beta transducin repeat‐containing protein (β‐TrCP), thereby enhancing T cell viability.^[^ ^41^ ^]^ Among them, the glycosylation of N192, N200 and N219 contributes to the stability of PD‐L1 protein by antagonizing the binding of PD‐L1 to GSK3β.^[^ ^41^ ^]^ Gao et al. also found that acetylation‐dependent regulation of PD‐L1 nuclear translocation enhances the anti‐cancer efficacy of PD‐1/PD‐L1 blockade.^[^ ^42^ ^]^ During the last decade, accumulating evidence has indicated that ubiquitination and deubiquitination of PD‐L1 play pivotal roles in the regulation of PD‐1/PD‐L1‐mediated immunosuppression, which suggests that targeting ubiquitin‐protein enzymes and deubiquitinating enzymes (DUBs) is a novel strategy to improve antitumor immune responses.^[^ ^43^, ^44^ ^]^ Several E3 ubiquitin ligases, such as SPOP, β‐TrCP, STIP1 homology and U‐box containing protein 1 (STUB1), and Ariadne‐1 homolog (ARIH1), have been reported to interact with PD‐L1 and mediate proteasome‐dependent degradation,^[^ ^32^, ^41^, ^45^, ^46^ ^]^ while DUBs such as CSN5, USP22, and USP9X can improve the stability of PD‐L1.^[^ ^47^ ^]^ SPOP functions as a tumor suppressor by promoting ubiquitination and degradation or functional regulation of carcinogenic proteins such as AR and BRD4.^[^ ^48^, ^49^ ^]^ Moreover, Zhang et al. revealed that SPOP inhibited the immune escape of cancer cells by degrading PD‐L1 in prostate cancer.^[^ ^32^ ^]^ In this study, we demonstrated that SRC‐1 could interact with SPOP but could not be degraded by SPOP, suggesting that SRC‐1 executes a sponge‐like function to absorb SPOP, leading to reduce the chances of the interaction between SPOP and PD‐L1, and consequently stabilize PD‐L1. Notably, SRC‐1 could be recruited to SPOP under normal conditions, and IFNγ treatment does not affect the amount of SRC‐1 recruited to SPOP (Figure S8A, Supporting Information), indicating that SRC‐1 can bind to SPOP to interfere the degradation of PD‐L1 protein by SPOP under both normal condition and IFNγ stimulation condition. We further investigate whether SRC‐1 can function through binding with other E3 ligases such as β‐TrCP, STUB1, and ARIH1 to interfere with the interaction of these E3 ligases to PD‐L1, and found that SRC‐1 could not interact with these E3 ligases (Figure S8B, Supporting Information). Taken together, these results indicate that SRC‐1 mainly improved the protein stability of PD‐L1 by binding with SPOP to interfere with the binding between SPOP and PD‐L1, rather than by binding with β‐TrCP, STUB1 or ARIH1.

In the absence of IFNγ stimulation, the transcription and protein levels of PD‐L1 are lower, and less SRC‐1 is enriched at the PD‐L1 promoter, therefore more SRC‐1 proteins are available to prevent SPOP‐mediated PD‐L1 protein degradation, in which case SRC‐1 is more important for the stability regulation of PD‐L1 protein. Under the condition of IFNγ stimulation, the transcription and protein levels of PD‐L1 were significantly elevated, and SRC‐1 was markedly enriched to the PD‐L1 promoter (Figure 4F; Figure S3D, Supporting Information); however, there was no significant change in SRC‐1 and SPOP protein levels, as well as the interaction between SRC‐1 and SPOP. These results indicate that transcriptional regulation of PD‐L1 by SRC‐1 plays a more important role upon IFNγ stimulation.

Since PD‐1, the receptor for PD‐L1, is one of the most potent suppressive molecules of T cells, inhibition of PD‐L1 expression by SRC‐1 deletion in CRC could activate T cells in TME to prevent tumor immune escape. Indeed, we observed that SRC‐1 deficiency increased the number of infiltrating CD3^+^ and CD8^+^ cells in graft mouse tumors. More importantly, the proportions of tumor‐infiltrating IFNγ^+^, GZMB^+^, and TNFα^+^ CD8^+^ T cells were significantly elevated after SRC‐1 deletion, indicating more effector CD8^+^ T cells were activated. In vitro cell killing experiments showed that SRC‐1 deletion in CRC cells rendered tumor cells more sensitive to be killed by CD8^+^ T cells, which could be reversed by rescued expression of PD‐L1 in SRC‐1‐deficient CRC cells. These results suggest that SRC‐1 deficiency promotes tumor infiltration and cytotoxicity of CD8^+^ T cells by reducing PD‐L1 expression. Clinically, SRC‐1 is frequently overexpressed in human CRC, which is positively correlated with PD‐L1 expression, but inversely correlated with the number of tumor‐infiltrating CD8^+^ T cells, implicating that targeting SRC‐1 may improve antitumor immunity.

Immune checkpoint inhibitor (ICI) is currently emerged as one of the most promising approaches to treat cancer, but it is still largely refractory to numerous patients.^[^ ^50^ ^]^ PD‐L1 antibody immunotherapy can indeed relieve the inhibition of CD8^+^ T cells, thereby releasing GZMB and cytokines (such as IFNγ and TNFα) to kill tumor cells. However, IFNγ and TNFα released by effector T cells in turn inhibit the immune surveillance to tumor cells by up‐regulating PD‐L1 expression in tumor cells, which is the main cause of insensitivity or drug resistance to anti‐PD‐1/PD‐L1 immunotherapy.^[^ ^50^, ^51^ ^]^ Our previous studies demonstrated that PD‐L1 blockade indeed up‐regulated the expression of PD‐L1 in tumor tissues, along with high levels of IFNγ and TNFα.^[^ ^8^ ^]^ Besides, this study found that PD‐L1 antibody monotherapy up‐regulated PD‐L1 expression by activating JAK1‐STAT3 signaling, which is one of the factors that makes PD‐L1 antibody monotherapy insensitive or resistance. Intriguingly, we observed that anti‐PD‐L1 therapy slightly increased the expression of SRC‐1 in tumor tissues, which may also be one of the reasons for the elevated PD‐L1 levels (Figure 6J). To further explore the mechanism of SRC‐1 up‐regulation, we treated CRC cells with different concentrations of IFNγ and TNFα, and found that high concentrations of IFNγ (500 ng mL^−1^) and TNFα (40 ng mL^−1^) could up‐regulate the expression of SRC‐1 (Figure S9, Supporting Information), indicating that αPD‐L1 treatment may induce SRC‐1 expression by promoting the production of different cytokines such as IFNγ and TNFα in the TME. However, Stattic significantly inhibited STAT3‐IRF1 signaling, but did not affect SRC‐1 expression (Figure 3D; Figure S2D, Supporting Information). These results indicate that multiple cytokines produced in the TME after αPD‐L1 treatment may be the key to the up‐regulation of SRC‐1.

Considering that monotherapy strategy cannot effectively prevent tumor immune evasion and metastasis, targeting therapy combined with immunotherapy strategies has attracted more and more attention. In the current study, we found that bufalin, a well‐established small molecule inhibitor of SRC‐1, combined with PD‐L1 antibody treatment in mice significantly reduced PD‐L1 expression and enhanced antitumor efficacy in CRC models, suggesting that bufalin improves the antitumor efficacy of PD‐L1 antibody treatment by down‐regulating PD‐L1 level, at least in part, by SRC‐1 in tumor cells. We recently found that PD‐L1 antibody treatment can activate multiple oncogenic pathways such as Wnt/β‐catenin, Hedgehog, and HIF1 in a JMJD2D‐dependent manner,^[^ ^8^ ^]^ which also contributes to the resistance of CRC to PD‐L1 antibody treatment. Since SRC‐1 can also activate Wnt/β‐catenin, Hedgehog, and HIF1 signaling pathways, targeting SRC‐1 by bufalin treatment may enhance the sensitivity of CRC to PD‐L1 antibody treatment by inhibiting these oncogenic pathways, too. In addition, studies have found that bufalin could act as a molecular glue, inducing E2F2 degradation through ZFP91 in an SRC‐1‐independent manner, thus exerting antitumor activity.^[^ ^52^ ^]^ Taken together, bufalin can inhibit the occurrence and development of tumors through multiple SRC‐1‐dependent and independent mechanisms. Our research suggests that bufalin can inhibit the JAK1‐STAT3 signaling pathway by inhibiting the expression of SRC‐1, thereby down‐regulating the expression of PD‐L1, inhibiting the immune escape of CRC, and ultimately exerting antitumor effects.

In summary, our study reveals that SRC‐1 promotes CRC immune escape by up‐regulating PD‐L1 expression via activation of JAK1‐STAT3 signaling and increase of PD‐L1 protein stability by preventing PD‐L1 interaction with SPOP. Furthermore, our study demonstrates that the combination therapy with SRC‐1 inhibitor and PD‐L1 antibody is much superior to monotherapy with either one, representing a novel strategy to potentiate immunotherapy in CRC patients with high levels of SRC‐1.

## 4 Experimental Section

### Cell lines

The murine CRC cell lines (CMT93, CT26, MC38), human CRC cell lines (HCT116) and human embryonic kidney cell line HEK‐293T were purchased from Cell Bank of Type Culture Collection of Chinese Academy of Sciences (Shanghai, China) and maintained in our lab. CMT93, HCT116 and HEK‐293T were maintained in Dulbecco's modified Eagle's medium (DMEM) medium (SH30045.05, HyClone) supplemented with 10% fetal bovine serum (FBS) (P30‐3302, PAN‐Biotech) and 100 U L^−1^ penicillin‐streptomycin. CT26 and MC38 were maintained in RPMI 1640 medium (SH30045.04, HyClone) supplemented with 10% FBS and 100 U L^−1^ penicillin‐streptomycin. All cells were maintained in an incubator with 5% CO_2_ at 37 °C.

### Mice

Male C57BL/6, BALB/c and nude mice were obtained from the Laboratory Animal Center of Xiamen University. OT‐I mice were kindly provided by Dr. Nengming Xiao's Lab and identified by FACS. The mice used in this study were six‐week‐old to eight‐week‐old. All mice experimental procedures performed here were approved by the Laboratory Animal Center of Xiamen University.

### Patients‐Derived Colorectal Tissue Samples

Tissue microarray analysis (TMA) containing seventy‐five paired human CRC tumors and matched adjacent non‐tumor tissues were obtained from Shanghai Outdo Biotech (Shanghai, China). Thirty‐six human CRC primary tumor specimens and matched adjacent normal tissues were collected from Zhongshan Hospital of Xiamen University (Xiamen, China). The specimens were surgically removed from patients, and informed consent was obtained from all patients.

### Stable Cell Lines Establishment

HA‐, Myc‐, Flag‐tagged plasmids for SRC‐1, STAT3, IRF1, SPOP, PD‐L1, β‐TrCP, STUB1, and ARIH1 were prepared according to standard molecular biology techniques. CMT93 and CT26 were infected with specific shRNA lentiviruses. The knockout plasmid of SRC‐1 used LentiCRISPR v2 as the vector, and the murine or human sgRNA designed by Zhang's laboratory online website was cloned into it, respectively.

The specific sequences were as follows: h‐SRC‐1‐1, 5′‐GATTCTACACCCGTAATAGC‐3′, h‐SRC‐1‐2, 5′‐ACTGCATTACTTCATAACGC‐3′, m‐SRC‐1‐1, 5′‐CTGCACGTCATCATCAGTCG‐3′, m‐SRC‐1‐2, 5′‐ATGCAGTATGCTGTAGACGC‐3′.

The shRNA of SRC‐1 was cloned into the pll3.7‐puro vector, the specific sequences were as follows: m‐SRC‐1‐1, 5′‐CAGCAGCTACTGACTGAATAA‐3′, m‐SRC‐1‐2, 5′‐CCCACCAAACTATGGTACAAA‐3′.

Lenti‐CRISPR v2‐puro vector, pll3.7‐puro vector or plasmid containing sgRNA or shRNA and equivalent packaging plasmids (PMDL, VSVG, and REV, 5:3:2) were co‐transfected into 293T cells, and the lentivirus was acquired after 48 h. CRC cells were infected with lentiviruses of their respective species and screened with puromycin to obtain stable knockout cells.

### siRNA‐Mediated SOCS1 or HDAC8 Knockdown

siRNAs targeting SOCS1 or HDAC8 were purchased from MiaoLingBio (Wuhan, China). Mouse SOCS1, HDAC8 or negative control siRNAs were transfected into CRC cells using Lipo6000 transfection reagent obtained from Beyotime Biotechnology according to the manufacturer's instructions (Shanghai, China).

### Real‐Time Quantitative PCR (RT‐qPCR)

The total RNA in cells and tissues was purified with TRIzol and extracted according to the manufacturer's instructions (15 596 018, Invitrogen). Total RNA was used to synthesize the cDNA using RT Master Mix (FSQ‐201, Toyobo). RT‐qPCR was performed by universal SYBR Green master mix using a Biorad CFX96 or CFX384 system (AQ131‐04, TransGen Biotech).

The sequences of RT‐qPCR primers were list as follows: h‐β‐actin, Forward 5′‐CATGTACGTTGCTATCCAGGC‐3′, Reverse 5′‐CTCCTTAATGTCACGCACGAT‐3′; h‐PD‐L1, Forward 5′‐TGGCATTTGCTGAACGCATTT‐3′, Reverse 5′‐TGCAGCCAGGTCTAATTGTTTT‐3′; h‐IRF1, Forward 5′‐GATGCTTCCACCTCTCACCAAGAAC‐3′, Reverse 5′‐ATGGCGACAGTGCTGGAGTCA‐3′; h‐SOCS1,

Forward 5′‐AACCTTCCTCCTCTTCCTCCTCCT‐3′, Reverse 5′‐AGTAGAATCCGCAGGCGTCCAG‐3′; h‐SPOP, Forward 5′‐GCCCCGTAGCTGAGAGTTG‐3′,

Reverse 5′‐ACTCGCAAACACCATTTCAGT‐3′; h‐HDAC8, Forward 5′‐ TGAATGCGTCTTCTACACCATCTC‐3′, Reverse 5′‐TTACGATTGCGACGGAAATTT‐3′; m‐β‐actin, Forward 5′‐ACTATTGGCAACGAGCGGTTCC‐3′, Reverse 5′‐GGCATAGAGGTCTTTACGGATGTCA‐3′; m‐PD‐L1, Forward 5′‐GTGGTGCGGACTACAAGCGAAT‐3′, Reverse 5′‐ACGGGTTGGTGGTCACTGTTTG‐3′; m‐IRF1,

Forward 5′‐CGAGGAAGTGAAGGATCAGAGTAGG‐3′, Reverse 5′‐GTGGTGTAACTGCTGTGGTCATCA‐3′; m‐SOCS1, Forward 5′‐CTGCGGCTTCTATTGGGGAC‐3′, Reverse 5′‐AAAAGGCAGTCGAAGGTCTCG‐3′; m‐SPOP, Forward 5′‐CCACCTCCGGCAGAAATGTC‐3′, Reverse 5′‐CCTCCCGGCAAAAACTAAAGT‐3′; m‐HDAC8, Forward 5′‐ ACTATTGCCGGAGATCCAATGT‐3′, Reverse 5′‐ CCTCCTAAAATCAGAGTTGCCAG‐3′; m‐IFNγ, Forward 5′‐ACTCAAGTGGCATAGATGTGGAAGA‐3′, Reverse 5′‐ATGACGCTTATGTTGTTGCTGATGG‐3′;

m‐TNFα, Forward 5′‐ACGTGGAACTGGCAGAAGAG‐3′, Reverse 5′‐GGTCTGGGCCATAGAACTGA‐3′; m‐Granzyme B, Forward 5′‐AGAACAGGAGAAGACCCAGCAAGT‐3′, Reverse 5′‐CCAACCAGCCACATAGCACACAT‐3′.

Relative expression of each gene was normalized against β‐actin and calculated by comparative threshold (Ct) cycle method (2^^‐ΔΔCt^).

### Western Blot

Cells or tissues were lysed with RIPA buffer supplementing with protease inhibitor cocktail and phosphatase inhibitors. The protein concentrations of lysates were quantified by BCA protein quantitative method. Equal quality protein was separated by SDS‐PAGE, and transferred to a PVDF membrane (Millipore). The PVDF membrane was cut according to protein molecular weight and incubated with the corresponding primary antibodies overnight at 4 °C. Then membranes were incubated with secondary antibodies. The signal was detected using ECL blotting substrates (Millipore) and captured by a chemiluminescence imager (Tanon, China). The primary antibodies used in western blot were list as follows: anti‐SRC‐1 (Cat# 2191S, CST), anti‐m‐PD‐L1 (Cat# 60 475, CST), anti‐h‐PD‐L1 (Cat# 13 684, CST), anti‐β‐actin (Cat# A5441, Sigma–Aldrich), anti‐IRF1 (Cat# 8478S, CST), anti‐SOCS1 (Cat# A7754, Abclonal), anti‐HDAC8 (Cat# 17548‐1‐AP, Proteintech), anti‐JAK1 (Cat# 50 996, CST), anti‐p‐JAK1 (Cat# 74 129, CST), anti‐STAT3 (Cat# 9139S, CST), anti‐p‐STAT3^Y705^ (Cat# ab76315, Abcam), anti‐SPOP (Cat# 16750‐1‐AP, Proteintech), anti‐p65 (Cat# 8242S, CST), anti‐p‐p65 (Cat# 3033S, CST), anti‐Myc‐tag (Cat# 05–724, Sigma), anti‐HA‐tag (Cat# H9658, Sigma), anti‐Flag‐tag (Cat# F1804‐1MG, Sigma), anti‐GST‐tag (Cat# AE077, Abclonal), anti‐Ubiquitin (Cat# sc‐8017, Santa Cruz).

### Luciferase Activity Measurement

Cells were co‐transfected with corresponding plasmids, including reporter plasmid, indicated plasmids, and Renilla luciferase plasmid. The Renilla luciferase plasmid was acted as an internal control. 48 h after transfection, cells were harvested with lysis buffer, and luciferase activities were determined by the luciferase reporter assay system (E2820, Promega). Luciferase activities were normalized by Renilla luciferase activity.

### Co‐Immunoprecipitation (Co‐IP) Assay

Exogenous Co‐IP: co‐transfected target proteins with different tags in 293T cells, and extracted the proteins 48 h after transfection; Endogenous Co‐IP: directly collected the protein of CMT93 and HCT116 cells. Added an appropriate amount of lysis buffer to extract the protein, of which 5% was used as input. Co‐incubated the remaining lysate with Protein A/G Plus‐Agarose for 1 h at 4 °C. Wash the pellet three times with lysis buffer and once with wash buffer. Then, the sample was co‐incubated with indicated antibodies and Protein A/G Plus‐Agarose at 4 °C overnight. The co‐precipitates were dissociated from the beads and separated by SDS‐PAGE and blotted with specific antibodies.

### GST Pull‐Down Assay

GST pull‐down assay was performed as described previously.^[^ ^53^ ^]^ Briefly, the target sequence was ligated to a GST‐tagged prokaryotic expression plasmid, and protein expression was induced at low temperature with 1 mM isopropyl‐β‐D‐thiogalactopyranoside (IPTG). Incubated an appropriate amount of GST‐fused full‐length or fragmented target protein with GST‐beads, and then co‐incubated the obtained GST‐fused protein and the recombinant protein expressed by 293T cells overnight at 4 °C. The bound proteins were eluted and visualized by Western blotting using the indicated antibodies.

### Chromatin Immunoprecipitation (ChIP) Assay

ChIP assay was performed as described previously. Briefly, cells were cross‐linked with 1% formaldehyde for 10 min at room temperature (RT) and then terminated with 0.125 M glycine. The chromatin was immunoprecipitated with specific antibodies. ChIP DNAs were purified and detected by RT‐qPCR using the promoter primers of IRF1 and PD‐L1. The ChIP primers used in this study were listed as follows:

h‐PD‐L1 promoter (STAT3 binding site), Forward 5′‐AGACCTCAAGAGTCATGATGAACTA‐3′, Reverse 5′‐ACACACACATGTACAACAAGTTTCA‐3′; h‐PD‐L1 promoter (IRF1 binding site), Forward 5′‐TTAATCTTCGAAACTCTTCCCGGTG‐3′, Reverse 5′‐CCTCTGCCCAAGGCAGCAAATCCAG‐3′; h‐IRF1 promoter,

Forward 5′‐CCGAATTAATAAGAGCCTACAGGAG‐3′, Reverse5′‐TATGCTTATTATGACTAGGGGTGCT‐3′; m‐PD‐L1 promoter (STAT3 binding site), Forward 5′‐CTGCAGGTAAGGGAGCATCTTCTCG‐3′, Reverse 5′‐CAGTCAAGTCGCGCTAGGACCAATT‐3′; m‐PD‐L1 promoter (IRF1 binding site), Forward 5′‐TTACTCTGGACTGTTTCTTTGAGGG‐3′, Reverse 5′‐CTTAATTCCAGTACTCAGGAGCCAG‐3′; m‐IRF1 promoter, Forward 5′‐AAGATACCAACCAAGCTTTCAGACT‐3′, Reverse 5′‐AACTGAGCTACATCTTCAGCTAGGC‐3′.

### RNA‐Seq Analysis

Total RNA was extracted from the IFNγ‐induced Ctrl and SRC‐1‐deficient MC38 cells. mRNA was purified using oligo(dT)‐attached magnetic beads, and fragmented into small pieces using fragmentation reagent. First‐strand cDNA was generated using random hexamer‐primed reverse transcription, followed by a second‐strand cDNA synthesis. The synthesized double‐stranded cDNA was subjected to end‐repair and 3′ adenylated, and adapters were added to the ends of these cDNA fragments. Amplified the cDNA fragments, and the PCR products were purified with AMPure XP Beads. Fragment size and concentration were determined using the Agilent 2100 Bioanalyzer. Denatured the PCR product to single‐stranded, and circularized by the splint oligo sequence to obtain the final library. The library was amplified with phi29 to make DNA nanoball (DNB) and load into the patterned nanoarray, thereby sequencing by combinatorial Probe‐Anchor Synthesis (cPAS) (BGI‐Shenzhen, China).

### OT‐I CD8^+^ T Cells Isolation and Co‐Culture with Tumor Cells

Splenocytes were isolated from OT‐I mice. Gently homogenize spleen and pass through a 70 µm Nylon cell strainer. Lyse red blood cells for 5 min at RT and suspend in RPMI 1640 medium. Splenic OT‐I cells were magnetically purified by anti‐mouse CD8 microbeads (130‐117‐044, Miltenyi). Splenic cells were activated in RPMI 1640 medium containing 50 ng mL^−1^ ovalbumin (OVA)^257–264^ peptides, 10 ng mL^−1^ mouse recombinant IL‐2, and 50 µM 2‐mercaptoethanol. Ctrl and SRC‐1‐deficient tumor cells were pretreated with OVA peptides (2 µg mL^−1^) for 2 h. To assess tumoricidal activity of T cells, activated OT‐I CD8^+^ T cells were co‐cultured with tumor cells at a 1:10 ratio for 24 h. All cells were collected by trypsinization and analyzed by FACS.

### Immune Cell Isolation and Fluorescence Activated Cell Sorting (FACS)

Tumor tissues were minced in RPMI 1640 basal medium and digested with collagenase buffer (0.5 mg mL^−1^ collagenase IV and 1 mg mL^−1^ DNase I) at 37 °C for 30 min. Digestion was terminated by an equal volume of RPMI 1640 medium containing 10% FBS. The digested tissues were passed through a 70 µm cell strainer to obtain single cell suspension. Cells were incubated with the corresponding fluorophore conjugated antibody for 30 min in the dark at 4 °C. Zombie UV Fixable Viability Kit (Cat# 423 108, Biolegend) was selected to exclude dead cells from analysis. For intracellular cytokine staining, cells were induced by conditioned medium, including 50 ng mL^−1^ PMA (Cat# P1585, Sigma), 1 µg mL^−1^ Ionomycin (Cat# I139530, Aladdin) and GolgiPlug (Cat# 51–2301kz, BD Biosciences), for 6 h before staining with antibodies. After staining with relevant antibodies on the cell surface, cells were fixed and permeabilized using fixation and permeabilization solution (Cat# 51–2090kz, BD Biosciences) for 30 min. The samples were detected by BD Fortessa X‐20 flow cytometer and the data were analyzed by Flowjo X software. The fluorescence conjugated antibodies used in this study were listed as follows: PE‐Cyanine7 PD‐L1 (Cat# 155 406, BioLegend), APC/Cy7 CD45 (Cat# 103 116, BioLegend), FITC CD3 (Cat# 100 305, BioLegend), BV421 CD8 (Cat# 48‐0081‐82, Thermofisher), PE‐Cyanine7 CD8 (Cat# 25‐0081‐82, Thermofisher), PE CD8 (Cat# 326 019, BioLegend), BV510 CD4 (Cat# 100 559, BioLegend), APC/Cy7 CD4 (Cat# 235 487, BioLegend), APC CD4 (Cat# 17‐0041‐82, Thermofisher), APC IFNγ (Cat# 505 809, BioLegend), Percp/Cy5.5 TNFα (Cat# 506 321, BioLegend), PE GranzymeB (Cat# 347 091, BioLegend), Percp/Cy5.5 CD45R (Cat# 308 915, BioLegend).

For cell cycle analysis, cell pellets were collected and fixed in 75% ethanol at 4 °C overnight. Cells were first incubated with RNase A (20 µg mL^−1^) at 37 °C for 30 min to fully degrade intracellular RNA, and then stained with PI. Samples were analyzed by NovoCyte Quanteon flow cytometer.

### Immunohistochemistry (IHC)

Tissue samples were fixed with 4% formalin fixation and embedded in paraffin. Sectioned the paraffin‐embedded tissues to 5 µm thickness using a Leica paraffin microtome. The tissue section slides were first subjected to antigen retrieval, followed by IHC staining according to the method described by IHC kit (SP9000, ZSGB‐BIO). Tissue sections were then incubated with primary antibodies (m‐CD3, m‐CD8) and biotinylated secondary antibodies, which signal was detected by DAB reagent.

### Graft Tumor Model

Digested the CRC cells in a good growth state, counted and adjusted the concentration to 2*10^7^ cells mL^−1^. A total of 2*10^6^ cells were subcutaneously injected on the dorsal flanks of healthy mice. Mice were grown in a suitable environment with free access to water and food. Mouse body weight and tumor size were measured 7 days after subcutaneous injection. The volume of the tumor was calculated according to the following formula: Volume = Length*Width^2^*0.52. After three weeks, the mice were sacrificed, and tumor tissues were obtained for subsequent experiments. Besides, 7 days after subcutaneous inoculation in mice, the SRC‐1 inhibitor bufalin and PD‐L1 antibody (BE0101, BioXCell) were intraperitoneally injected every day and every two days, respectively.

### AOM/DSS‐Induced Mouse CRC Orthotopic Model

Six‐ to eight‐week‐old male wild‐type and *SRC‐1* ^−/−^ mice were intraperitoneally injected with azoxymethane (AOM) (10 mg kg^−1^). After one week, the mice were fed with 2% DSS for one week, then returned to normal drinking water for two weeks, and the process was repeated three times. The mice were sacrificed after 4 weeks, the colorectal tissues were stripped, photographed, and the number and size of tumors in the colorectal were counted. The colorectal tissues were awarded for subsequent experiments.

### Statistical Analysis

Statistical analyses were performed by GraphPad Prism 8 software (SanDiego, USA). Quantitative data were reported as ± SEM (standard error of the mean) with at least three replicates. Statistical significance was calculated by Student's t‐test, Pearson r tests and ANOVA. *p* value < 0.05 was regarded as statistically significant difference.

## Conflict of Interest

The authors declare no conflict of interest.

## Author Contributions

C.Y. and Y.Y. conceived and coordinated the study. Y.H., Q.C., Y.Y., and C.Y. conceived and designed the experiments. Y.H., Q.C., Z.W., Y.Z., B.L., H.G., C.H., and X.K. performed or analyzed the experiments. Y.H., Q.C., P.M., N.X., J.X., Y.Y., and C.Y. wrote the manuscript.

## Supporting information

Supporting Information

## Data Availability

The data that support the findings of this study are available from the corresponding author upon reasonable request.
