# Supplementary material for: Targeting Nuclear Receptor Coactivator SRC‐1 Prevents Colorectal Cancer Immune Escape by Reducing Transcription and Protein Stability of PD‐L1
Source: Adv Sci (Weinh). 2024 Jul 2;11(33):2310037. doi: 10.1002/advs.202310037 (PMC11434141; doi:10.1002/advs.202310037)
Supplement: Supplementary file 1 — Supporting Information [file ADVS-11-2310037-s001.pdf]

ADVANCED SCIENCE
Open Access

# Supporting Information

for *Adv. Sci.*, DOI 10.1002/advs.202310037

Targeting Nuclear Receptor Coactivator SRC-1 Prevents Colorectal Cancer Immune Escape by Reducing Transcription and Protein Stability of PD-L1

*Yilin Hong, Qiang Chen, Zinan Wang, Yong Zhang, Bei Li, Hanshi Guo, Chuanzhong Huang, Xu Kong, Pingli Mo, Nengming Xiao, Jianming Xu, Yunbin Ye* and Chundong Yu**

## Figure S1

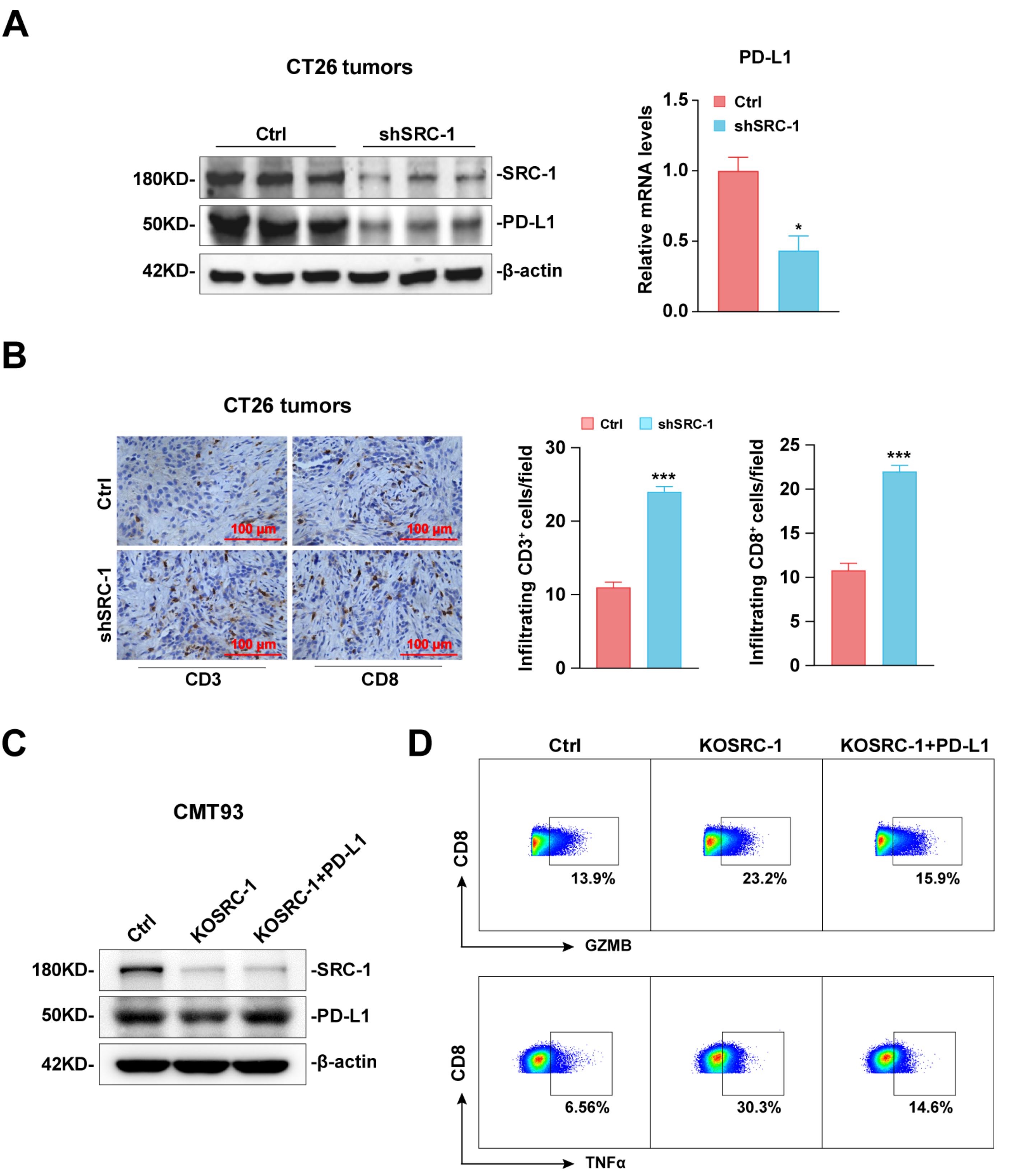


**Figure S1.**

Tumor cell-expressed SRC-1 reduces tumor infiltration and antitumor activity of $CD8^+$ T cells by regulating PD-L1. A) The protein and mRNA (*n*=4 per group) levels of PD-L1 were attenuated in SRC-1-deficient CT26 tumors. B) Knockdown of SRC-1 increased the number of infiltrated $CD3^+$ and $CD8^+$ T cells in CT26 tumors (*n*=5 per group). C) Exogenous expression of PD-L1 in SRC-1-deficient CMT93 cells. D) The effector function of $CD8^+$ T cells on exogenous PD-L1 expression in SRC-1-deficient CMT93 cells was reduced compared with SRC-1-deficient group (*n*=3 per group). Data are shown as mean ± SEM. *$p$<0.05, ***$p$<0.001, based on Student's t test.

# Figure S2

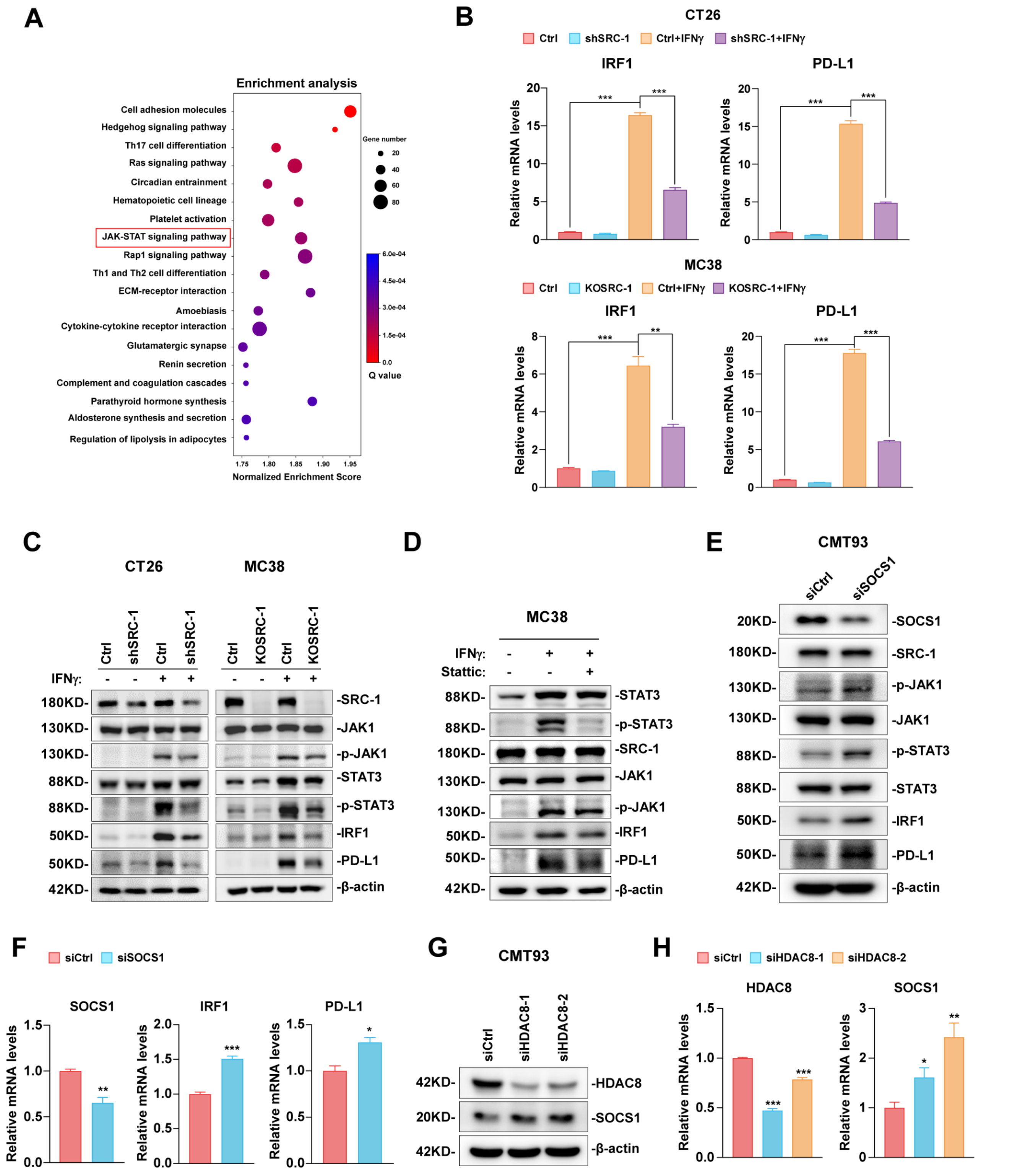


**Figure S2.**

Tumor cell-expressed SRC-1 modulates PD-L1 expression by promoting JAK1-STAT3-IRF1 signaling. A) GSEA identifies JAK-STAT signaling pathway as one of the top KEGG pathways. B) The mRNA levels of IRF1 and PD-L1 induced by IFNγ were decreased in SRC-1-deficient CT26 and MC38 cells (*n*=3 per group). C) Knockdown or knockout of SRC-1 down-regulated IFNγ-induced phosphorylation levels of JAK1 and STAT3, as well as protein levels of IRF1 and PD-L1 in CT26 and MC38 cells. D) Stattic decreased the expression of IRF1 and PD-L1 in MC38 cells. E, F) Knockdown of SOCS1 up-regulated the phosphorylation of JAK1 and STAT3, as well as protein and mRNA (*n*=3 per group) levels of IRF1 and PD-L1. G, H) Knockdown of HDAC8 up-regulated the protein and mRNA (n=3 per group) levels of SOCS1 in CMT93 cells. Results are representative of one of three experiments. Data are shown as mean ± SEM. *$p<0.05$, **$p<0.01$, ***$p<0.001$, based on Student's t test and one-way ANOVA.

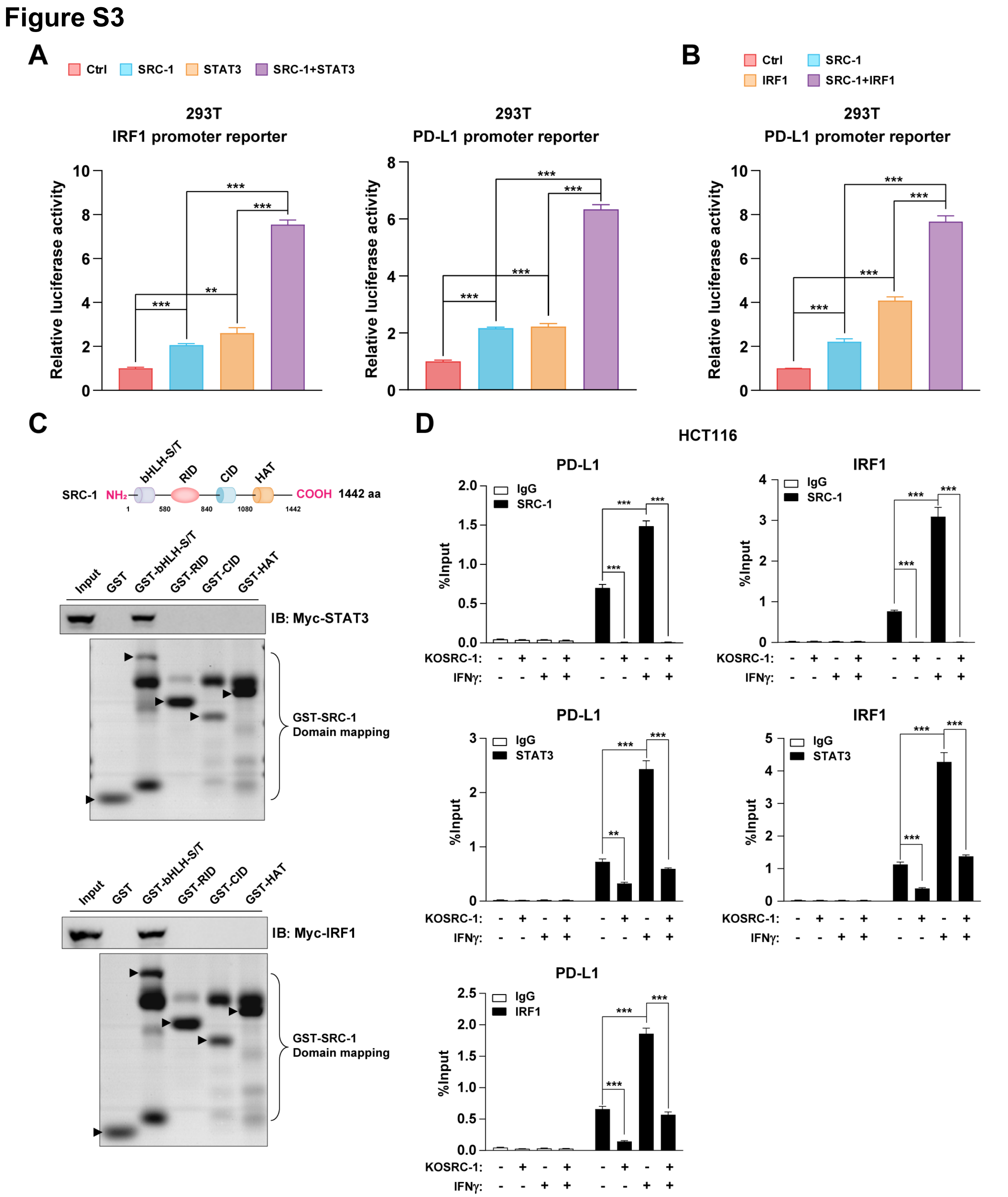


**Figure S3.**

SRC-1 cooperates with STAT3-IRF1 axis to promote PD-L1 transcription. A) SRC-1 cooperated with STAT3 to enhance the transcriptional activity of IRF1 and PD-L1 promoters in 293T cells (*n*=3 per group). B) SRC-1 cooperated with IRF1 to enhance the transcriptional activity of PD-L1 promoters in 293T cells (*n*=3 per group). C) Both STAT3 and IRF1 bound to the bHLH-S/T domain of SRC-1 detected by GST-pull down assays. The arrow shows the representation strip. D) The recruitment of IFNγ-driven STAT3 or IRF1 to their target gene promoters was prevented in SRC-1-deficient HCT116 cells (*n*=3 per group). Results are representative of one of three experiments. Data are presented as means ± SEM. **$p$<0.01, ***$p$<0.001, based on one-way ANOVA.

# Figure S4

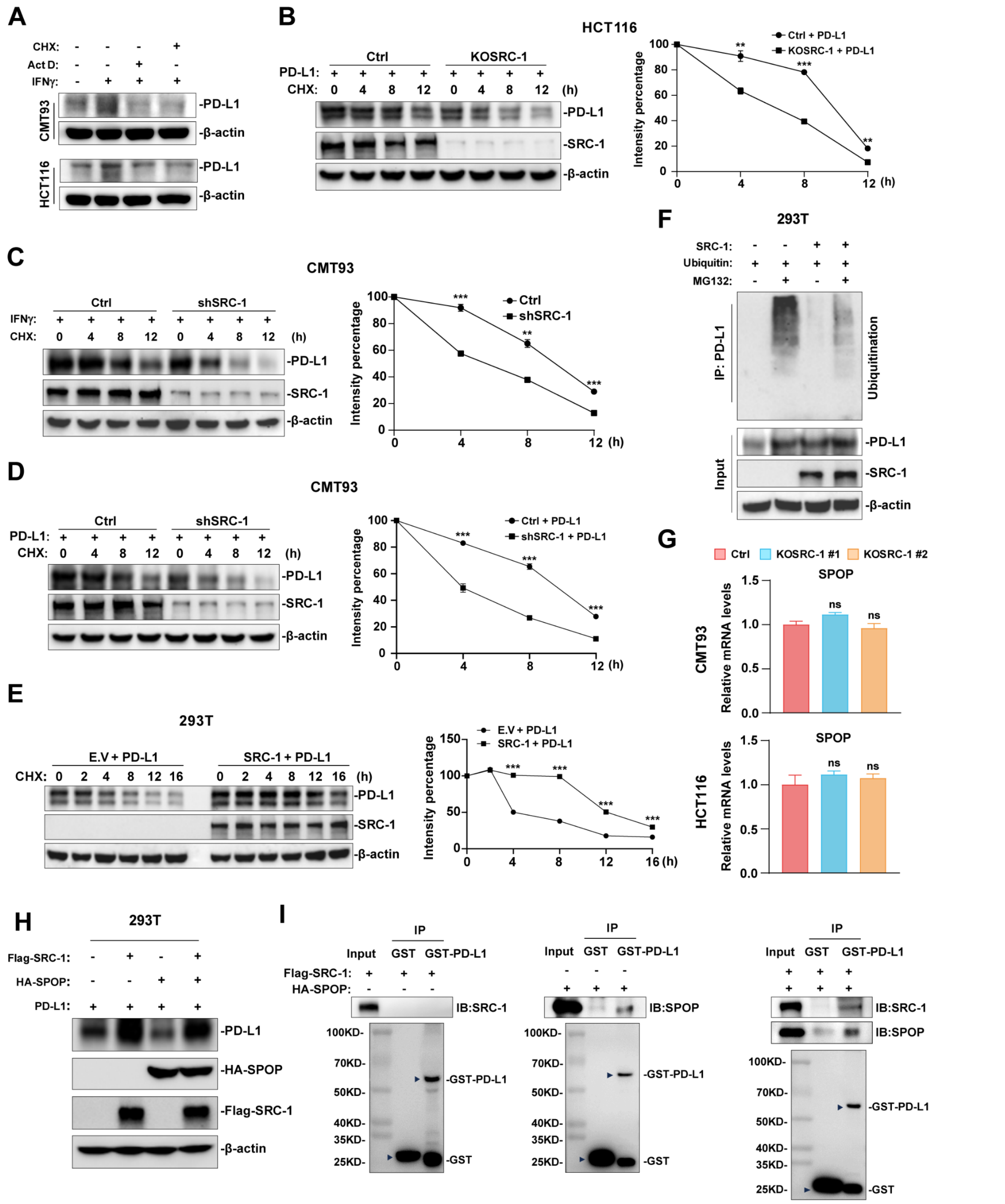


**Figure S4.**

SRC-1 stabilizes PD-L1 protein by inhibiting SPOP-mediated degradation. A) IFNγ-induced PD-L1 expression was attenuated in CMT93 and HCT116 cells treated with CHX or Act D. B) Knockout of SRC-1 promoted the degradation of exogenous PD-L1 protein in HCT116 cells. C) Knockdown of SRC-1 accelerated IFNγ-induced PD-L1 protein degradation in CMT93 cells. D) Knockdown of SRC-1 promoted the degradation of exogenous PD-L1 protein in CMT93 cells. E) SRC-1 stalled the degradation of exogenous PD-L1 protein in 293T cells. F) Proteasome inhibitor MG132-mediated the accumulation of ubiquitin-PD-L1 was reversed by exogenous SRC-1. G) Knockout of SRC-1 did not affect the mRNA level of SPOP in CMT93 and HCT116 cells (*n*=3 per group). H) SPOP-mediated PD-L1 degradation could be prevented by exogenous SRC-1 in 293T cells. I) SRC-1, SPOP, and PD-L1 can form SRC-1/SPOP/PD-L1 complexe in the presence of SPOP. Results are representative of one of three experiments. Data are presented as means ± SEM. $^{ns}p>0.05$, $^{**}p<0.01$, $^{***}p<0.001$, based on Student's t test and one-way ANOVA.

## Figure S5

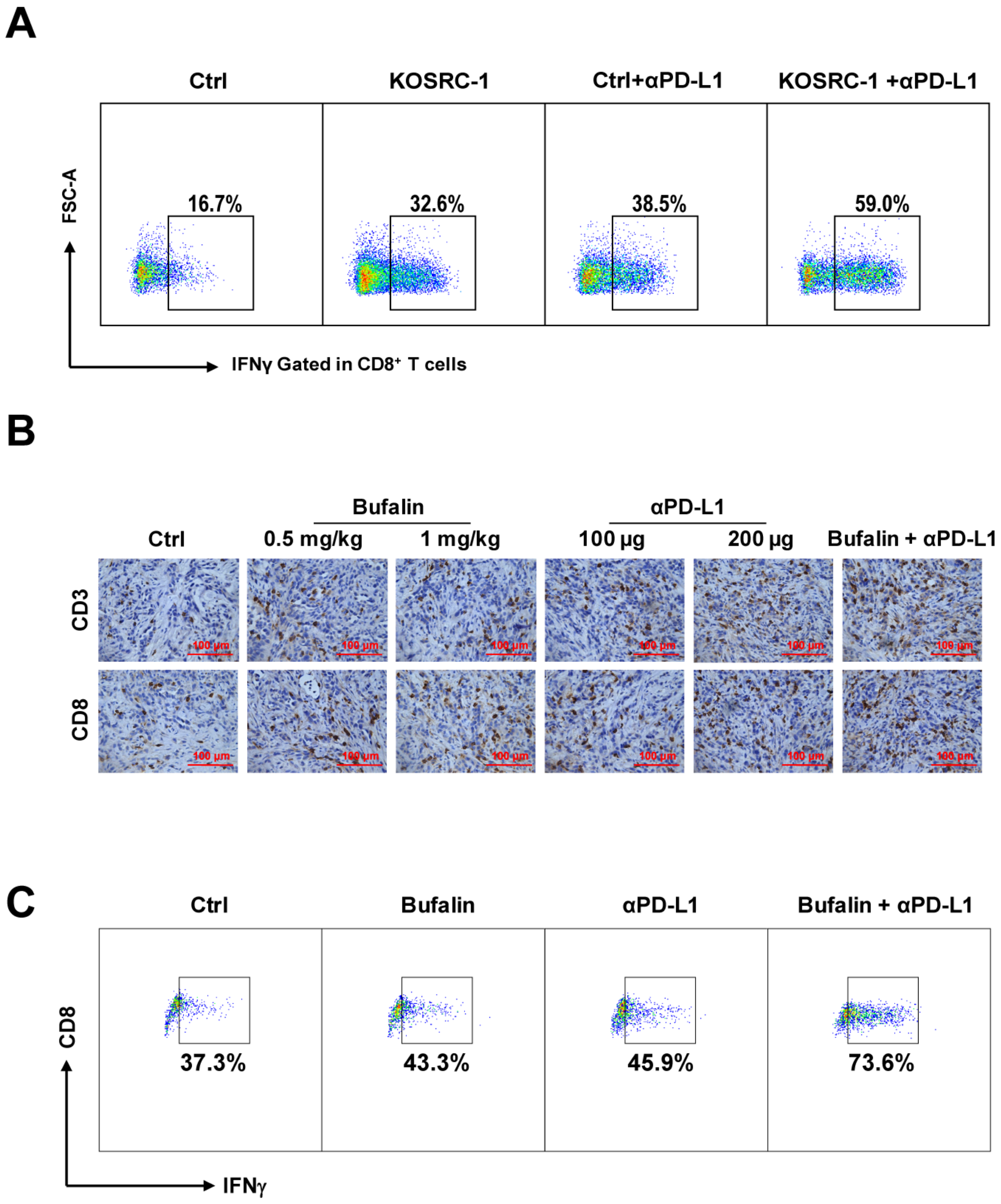


**Figure S5.**

Inhibition of SRC-1 improves the antitumor efficacy of PD-L1 antibody. A) Representative FACS analysis plots of IFNγ$^+$ cells among CD8$^+$ T cells in CMT93 tumors treated with SRC-1 knockout and PD-L1 antibody alone or in combination. B) Low dose combination treatment of bufalin and PD-L1 antibody increased the number of tumor-infiltrating CD3$^+$ and CD8$^+$ T cells in CMT93 tumors. C) Representative FACS analysis plots of IFNγ$^+$ cells among CD8$^+$ T cells in CMT93 tumors treated with bufalin and PD-L1 antibodies alone or in combination.

# Figure S6

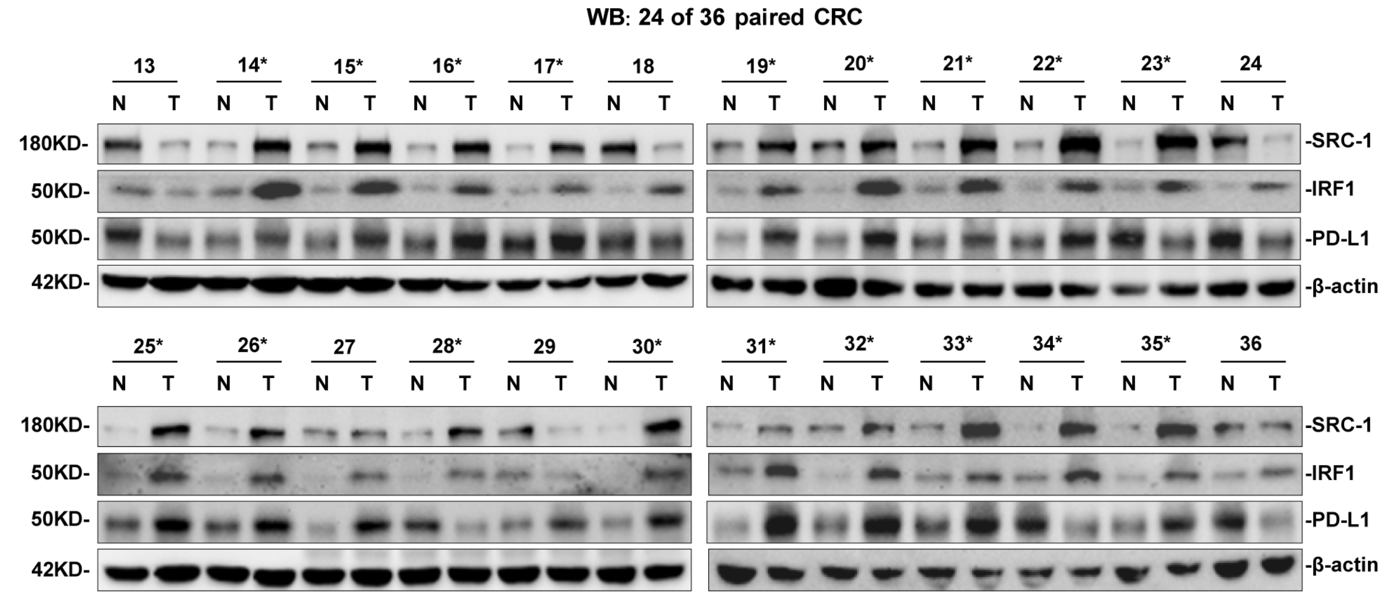


**Figure S6.**

SRC-1 and PD-L1 are frequently overexpressed in human CRC specimens. The protein levels of SRC-1, IRF1, and PD-L1 were frequently overexpressed in human CRC specimens (*n*=36). The asterisk indicates SRC-1-positive CRC specimens.

# Figure S7

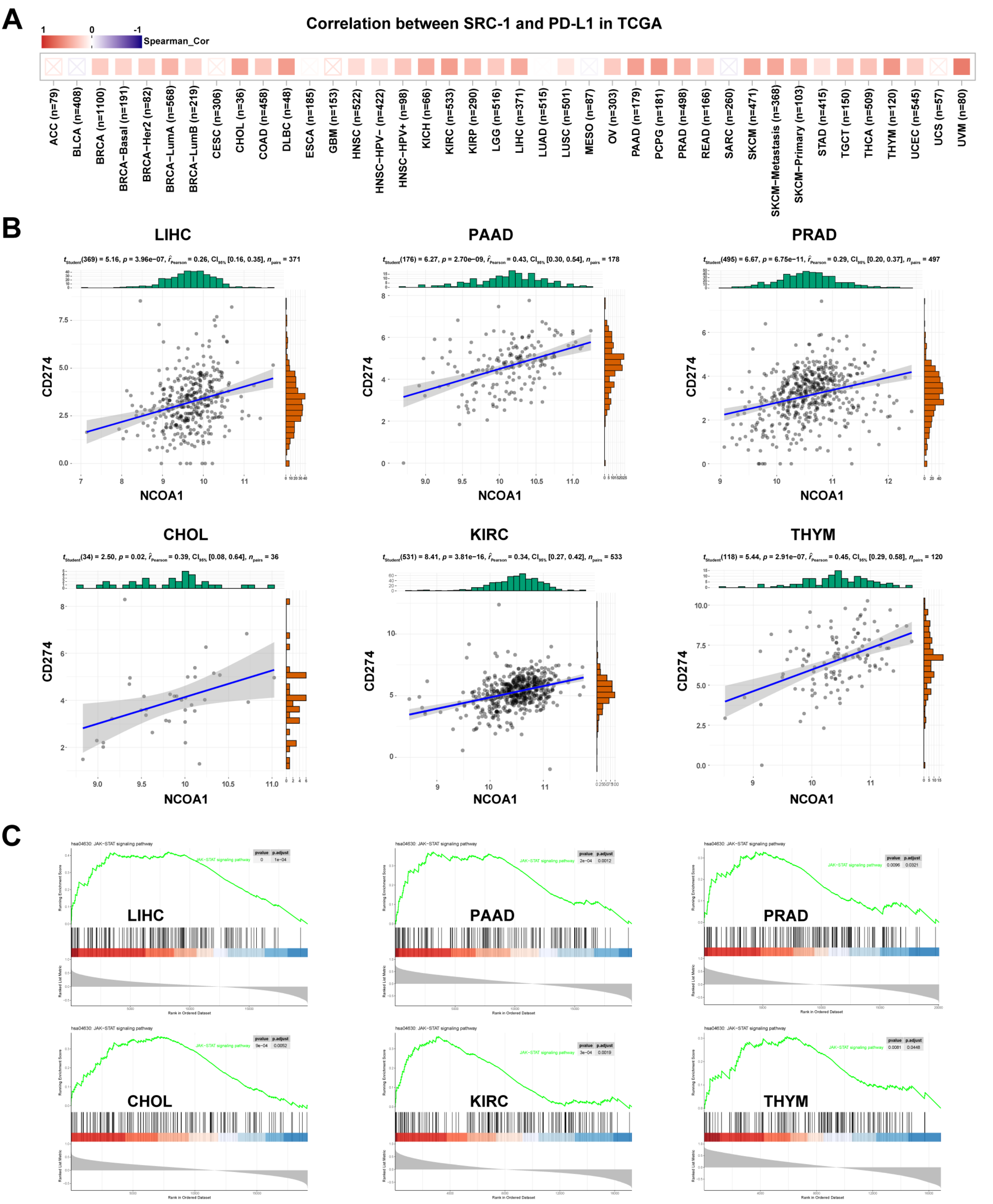


**Figure S7.**

SRC-1 was positively correlated with PD-L1 in numerous cancer cell panels. A) The positive correlation between SRC-1 and PD-L1 in CRC and numerous non-CRC cancer cell panels in TCGA database. B, C) The positive correlation between SRC-1 and PD-L1 and notable enrichment of JAK-STAT pathway were found in numerous non-CRC cancer cell panels, including LIHC, PAAD, PRAD, CHOL, KIRC and THYM.

## Figure S8

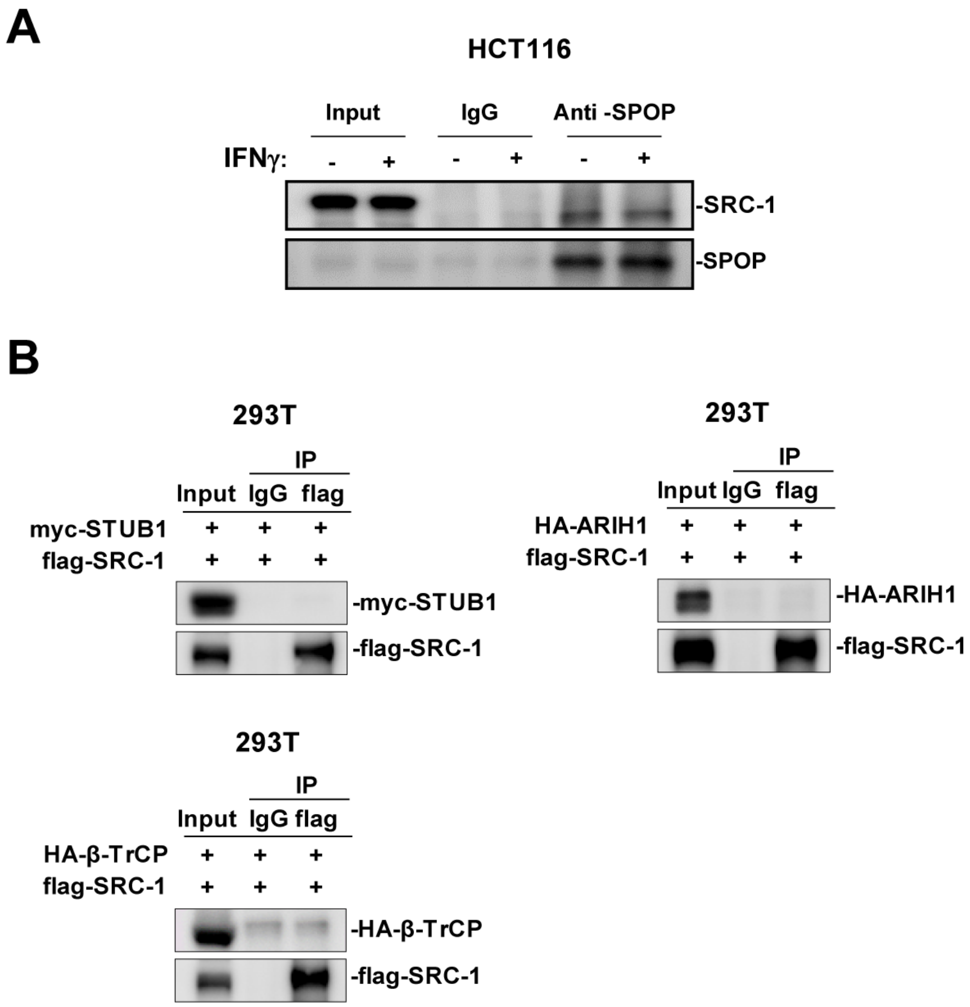


**Figure S8.**

SRC-1 interacts with the E3 ligase SPOP, but not with β-TrCP, STUB1, and ARIH1. A) IFNγ did not affect the interaction between SRC-1 and SPOP in HCT116 cells. B) SRC-1 does not interact with β-TrCP, STUB1, and ARIH1.

# Figure S9

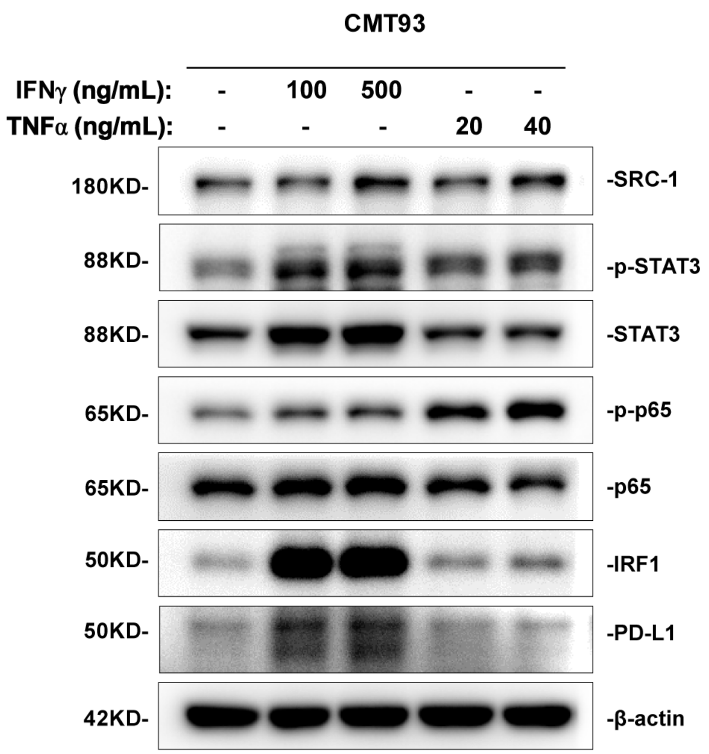


**Figure S9.**

High concentrations of IFNγ or TNFα induce SRC-1 expression in CRC cells.
